# Differential responses of selectively bred mussels (*Perna canaliculus*) to heat stress—survival, immunology, gene expression and microbiome diversity

**DOI:** 10.3389/fphys.2023.1265879

**Published:** 2024-02-15

**Authors:** Jessica A. Ericson, Olivier Laroche, Laura Biessy, Natalí J. Delorme, Xavier Pochon, Jacob Thomson-Laing, Norman L. C. Ragg, Kirsty F. Smith

**Affiliations:** ^1^ Cawthron Institute, Nelson, New Zealand; ^2^ Institute of Marine Science, University of Auckland, Auckland, New Zealand; ^3^ School of Biological Sciences, University of Auckland, Auckland, New Zealand

**Keywords:** transcriptomics, RNAseq, microbiome, green-lipped mussel, biomarkers, immune response

## Abstract

New Zealand’s green-lipped mussel (*Perna canaliculus*) is an ecologically and economically important species. Marine heatwaves are increasing in frequency around NZ’s coastline, and these events are correlated with increased stress and mortality of some aquaculture species. This study aimed to identify general biomarkers of heat stress in *P. canaliculus* and to assess whether responses differed between genetically distinct selectively bred mussels. We exposed three families of selectively bred mussels (families A, B and C) to three seawater temperature regimes in the laboratory: 1) a “control” treatment (ambient 12°C), 2) a 26°C heat challenge with a subsequent recovery period, and 3) a sustained 26°C heat challenge with no recovery. We investigated whether the survival, immune response (hemocyte concentration and viability, oxidative stress and total antioxidant capacity), hemocyte gene expression and gill microbiome differed between the families during the temperature challenges. In the sustained heat-stress treatment, family A had the highest survival rate (42% compared with 25% and 5% for families C and B, respectively). Gene expression levels significantly shifted during thermal stress and differed between families, with family A more dissimilar than families B and C. Family C had substantially more genes impacted by temperature treatment and timepoint than the other families, while family B had very little genes/pathways that responded to thermal stress. Genes related to heat shock proteins and immune responses (e.g., AIF1, CTSC, TOLL8, CASP9, FNTA, AHCY, CRYAB, PPIF) were upregulated in all families during heat stress. Microbiome species-richness differed between families before and during heat-stress, with family A having a distinctly different microbiome flora than the other families. Microbial diversity changed similarly in all families exposed to prolonged heat-stress, with species of *Vibrio* and *Campylobacter* increasing in these mussels. Our study highlights the use of non-lethal sampling of hemocytes as a diagnostic tool to explore the immune response and gene expression of selectively bred mussels, to predict their response to ocean warming. This approach can identify potential thermotolerant candidates for further selective breeding, which may increase the resilience of the mussel aquaculture industry in a warming ocean.

## 1 Introduction

Marine heatwaves are increasing in intensity due to anthropogenic climate change, and have doubled in frequency over the past 30 years (IPCC, 2019). The waters around Aotearoa New Zealand and in the Tasman Sea are warming at nearly four times the global average rate (Oliver et al., 2017). Coastal waters are particularly vulnerable to these heatwaves and other climate change stressors (e.g., ocean acidification), in combination with pressure from terrestrial activities (e.g., runoff and sedimentation from agriculture and forestry). Marine farms around the world are primarily located in coastal regions, where they are easily accessible from land and more sheltered from large swells. The effects of climate change on aquaculture can be positive, neutral or negative, depending on the region and the species being farmed (Maulu et al., 2021). Inshore operations are often more vulnerable to coastal ocean acidification and heatwaves, which has resulted in numerous mass mortality events of farmed bivalves, especially during summer (Masanja et al., 2023).

The green-lipped mussel (*Perna canaliculus*) is a culturally and ecologically significant bivalve endemic to Aotearoa New Zealand. It is also Aotearoa New Zealand’s most valuable aquaculture species, contributing over $300 M NZD per annum to the economy (Miller et al., 2023). Like other aquaculture species around the world, mortalities of farmed green-lipped mussels are occurring in some regions during summer (Newton and Webb, 2019; Li et al., 2020; Nguyen and Alfaro, 2020). If ocean waters surrounding New Zealand continue to warm as predicted (Salinger et al., 2019; de Burgh-Day et al., 2022), these mortality events are likely to increase in frequency. Current mortality events are often associated with above average seawater temperatures and in some cases, infection with bacterial species such as *Vibrio* spp./*Vibrio*-like spp. (Newton and Webb, 2019; Nguyen and Alfaro, 2020; Azizan et al., 2022a; Ericson et al., 2022). Numerous recent studies have investigated the effects of elevated seawater temperatures on the physiology of green-lipped mussels, to understand the mechanisms that lead to decreased health and survival of these mussels during heat-stress (Dunphy et al., 2015; Li et al., 2020; Nguyen and Alfaro, 2020; Ericson et al., 2022; Ericson et al., 2023a; Azizan et al., 2023; Ericson et al., 2023b; Venter et al., 2023). These studies have shown that mussels respond to heat stress via the activation of metabolic pathways controlling energy metabolism and antioxidant production (Delorme et al., 2021; Azizan et al., 2023). The immune response of green-lipped mussels varies depending on the severity of heat stress. Percentages of non-viable and superoxide positive hemocytes increase markedly during extreme acute heat stress (e.g., >28.5°C immersion for <60 min; Delorme et al., 2021), but effects are more subtle and complex during chronic heat stress at lower temperatures (e.g., 24°C immersion for weeks to months; Ericson et al., 2022; Ericson et al., 2023b). Field-based studies demonstrated strong evidence of changes to energy and immune-related metabolic pathways in stressed mussels (Li et al., 2020; Nguyen and Alfaro, 2020). An organism’s ability to recover from heat-stress depends on the duration of the temperature challenge and the integrated thermal history experienced before a given heatwave event (Siegle et al., 2018). Seawater temperatures of 26°C have been identified as a survival “tipping point” for green-lipped mussels, where mortality begins after several days at this temperature (Ericson et al., 2023a).

The molecular response of marine invertebrates to environmental stress has been well-studied (see reviews: Gleason, 2019; Veldhoen et al., 2012; Page and Lawley, 2022) but to date, genomic and transcriptomic resources have been limited for *P. canaliculus* (but see Baettig et al., 2023a). High-throughput sequencing approaches (e.g., RNAseq) can measure the expression levels for all genes within a single sample, even for non-model organisms. These techniques now enable an understanding of molecular mechanisms at the individual level including in response to stress (Ekblom and Galindo, 2011). Field-based studies have demonstrated strong evidence of changes to energy and immune-related metabolic pathways in stressed mussels (Li et al., 2020; Nguyen and Alfaro, 2020). Gene transcription is the first step in the synthesis of gene products (e.g., proteins) and alterations to gene expression capture the effects on organisms at the lowest level of biological organization—the cell or tissue type. These can occur very rapidly, meaning the response to stressors can be detected at the earliest stage. This allows the development of sensitive biomarkers that target the expression levels of specific genes that can be diagnostic of a stressor before a lethal event occurs, enabling mitigation measures to be invoked to avoid mortality (Louis et al., 2017).

The microbiota of marine invertebrates is also known to play a crucial role in host adaptation (Laroche et al., 2018; Wilkins et al., 2019; Marangon et al., 2021; Liu et al., 2023). These microbial communities offer their host organisms various beneficial functions including pathogen defense, immunological regulation, and improved digestion efficiency and nutrient uptake. Microbiome communities are therefore expected to undergo changes when the host adapts to stressful conditions. Characterizing the microbial structure of marine invertebrates can determine host-microbiome interactions and inform on the health status of the host organism (Li et al., 2022).

Primary industries such as aquaculture need to implement mitigation measures if they are to adapt to food production in a changing climate (Maulu et al., 2021). Such measures may include diversification (e.g., growing more resilient species, moving to colder waters), utilization of indigenous knowledge and breeding for resilience (Maulu et al., 2021). Selective breeding has long been used in aquaculture to achieve more desirable traits in farmed stock (Boudry et al., 2021; Regan et al., 2021). Traditionally, these desirable traits have primarily related to variables such as shell color for bivalves, fast growth, disease resistance or feed efficiency (Gjedrem et al., 2012; Hollenbeck and Johnston, 2018). As the risks associated with climate change are now well documented, selective breeding for environmental resilience (e.g., thermal tolerance) has been identified as a key trait to include in selective breeding programmes (Pernet and Browman, 2021; Liu et al., 2022; Masanja et al., 2023). The use of selectively bred mussels in transcriptomic and microbiome studies also allows assessment of their response to different stressors while controlling for genetic variation, which can often confound such studies.

Our study had two main aims—1) To investigate general genomic, microbiomic and immune markers of heat stress in green-lipped mussels, and 2) To explore whether selectively bred families respond differently to heat-stress, and if so, to identify whether any differences between families could be used to identify potential resilient candidates for selective breeding. We investigated the response of three selectively bred mussel families to a range of thermal regimes in the laboratory: 1) a “control” treatment (12°C ambient temperature at the time of the experiment), 2) a “ramp and recover” treatment where seawater temperature was increased 1°C per day up to 26°C and then decreased at the same rate back down to 12°C (to allow recovery), and 3) a “ramp and hold” treatment where seawater temperature was increased 1°C per day up to 26°C and held there for 2-weeks. This research highlights the potential use of non-lethal samples (e.g., hemolymph samples) as diagnostic tools to inform selective breeding programmes. It is also the first published study to investigate the effects of heat-stress on the green-lipped mussel whole transcriptome and associated microbiome.

## 2 Materials and methods

### 2.1 Animal husbandry, temperature trajectories and water quality

Adult mussels from a commercial selective breeding programme were collected from a marine farm (Sanford Ltd.) in Pelorus Sound, Aotearoa New Zealand, and transported to the Cawthron Aquaculture Park (Nelson) in June 2021 during austral winter (ambient temperature at time of collection = 12°C). Mussels were collected from three genetically distinct families (hereafter A, B and C) that were the same age (3 years old), and 360 mussels were collected per family. These mussels were from the same 2018 breeding cohort and had been bred over two generations when these families were created. They were bred for traits relating to production and to create diversity within the commercial breeding programme, and were not bred for traits relating to heat tolerance. On average, Family A were smaller than families B and C (Family A shell length = 99.1 ± 8.2 mm (mean ± SD), Family B = 115.0 ± 8.0 mm, Family C = 116.9 ± 7.9 mm).

**FIGURE 1:**
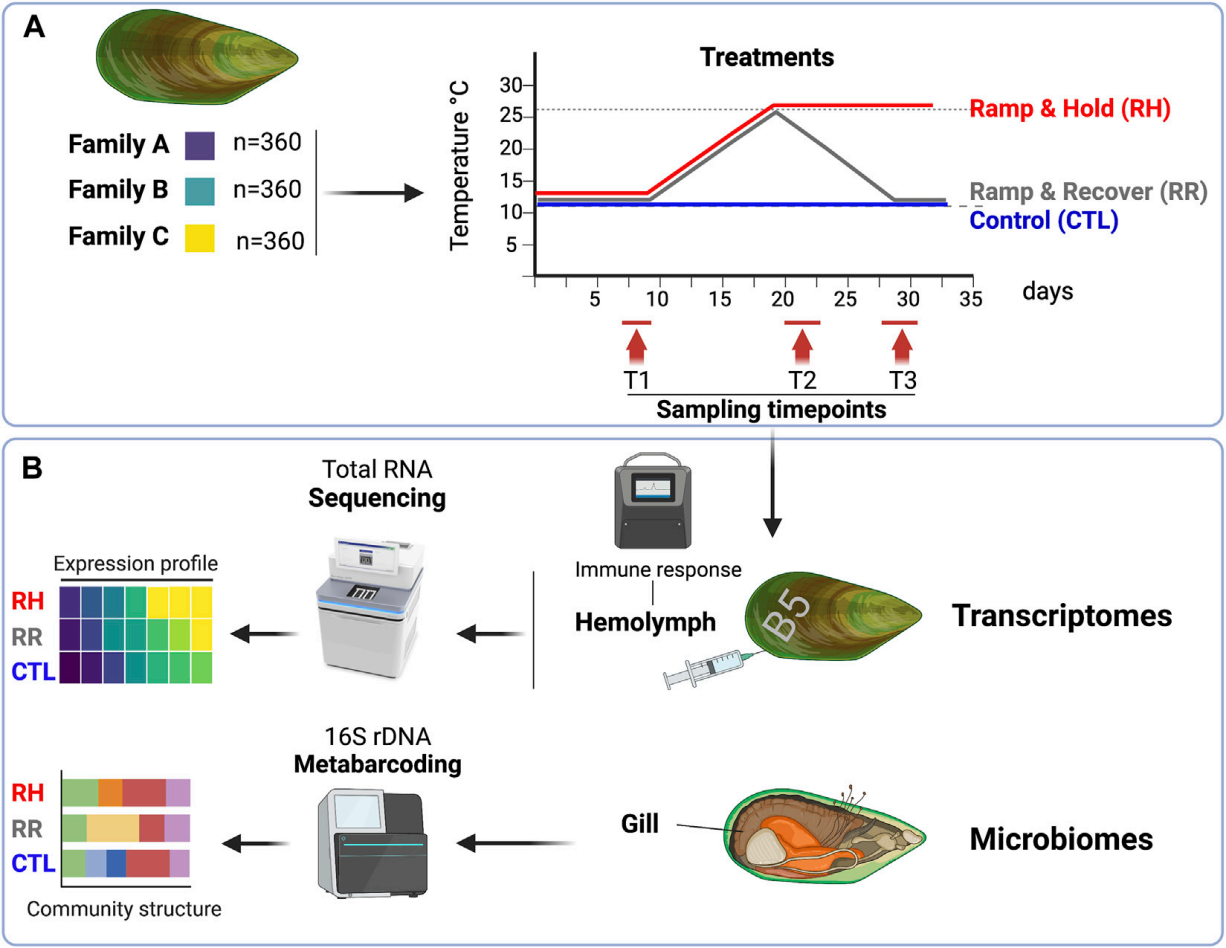
Experimental and analytical design. **(A)** Three genetically distinct green-lipped mussel families were subjected to three distinct temperature treatments over a 36-day period in June 2021, and sampled at three different timepoints (days 7–9 [T1]; days 20–22 [T2]; 28–30 [T3]. **(B)** At each sampling timepoint, the hemolymph from 16 pre-labelled mussels per family per treatment was collected for immune response and deep-RNA sequencing, and gill samples from five mussels per family per treatment were collected for microbiome analyses. Figure was created on Biorender.com.

Animals were engraved with a family identification code (either “A,” “B” or ‘C’) on arrival and placed into 18 tanks (20 animals per family per tank) in preparation for experimental trials (Figure 1A). Of the 20 animals per tank, two mussels per family were also engraved with a number identification so that they could be uniquely identified and used for repeated hemolymph sampling. All tanks were held at 12.3°C ± 0.3°C for 9 days to acclimate animals to the experimental system. Throughout the acclimation period and for the rest of the experimental trial, seawater was supplied from eutrophic algal ponds containing natural algal assemblages. Seawater was unfiltered to allow for some natural sediment to remain in the seawater.

After the 9-day acclimation period, mussels were exposed to three different temperature trajectories for the remainder of the experimental period (Figure 1A). The three temperature treatments were: 1) a “control (CTL)” treatment which remained at 12°C for the trial duration (*n* = 2 tanks), 2) a “ramp and recover (RR)” treatment where seawater temperatures were increased at an average rate of 1°C per day up to 26°C, then decreased at an average rate of 1.5°C per day down to 12°C (*n* = 8 tanks), and 3) a “ramp and hold (RH)” treatment where seawater temperatures were increased at an average rate of 1°C per day up to 26°C and held there (*n* = 8 tanks). Temperatures in both ramping treatments were staggered slightly to enable sampling to be carried out on separate days, but with the same ramping speed. Control tanks and heat-stress tanks all had the same water source, but control tanks were located in a separate room to the treatment tanks for logistical reasons.

Tank flow rates were measured twice daily and held at 2–3 L/min, and tank dissolved oxygen levels were measured daily using a dissolved oxygen meter (Oxyguard Polaris). Tanks were checked twice daily for mortalities (wide valve opening and no response after gentle mantle stimulus using a dissecting straightpoint needle). If mortalities were found the family identification number was recorded and the length of each dead mussel was measured using calipers and recorded.

### 2.2 Mussel gill and hemolymph sampling

**FIGURE 2:**
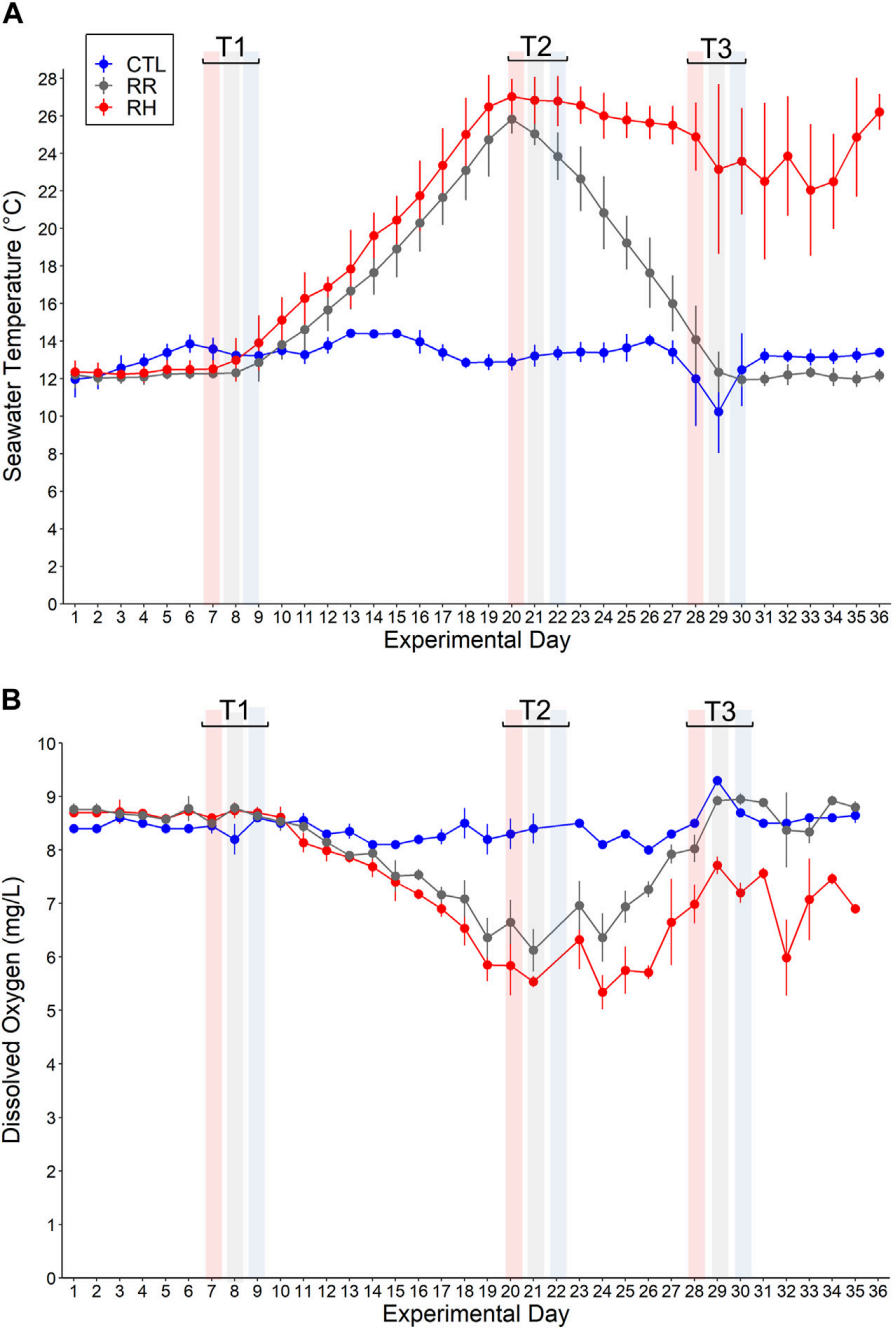
**(A)** Seawater temperature (°C) and **(B)** dissolved oxygen (mg/L) (mean ± SD) for each temperature treatment (RR, RH and CTL) on each experimental day 1–36. Sampling timepoints 1–3 (denoted as T1, T2 and T3) for each treatment are also shown.

Mussels from each treatment were sampled at three different sampling points (T1, T2 and T3) during the trial (see Figures 1A, 2 for the sampling days for each treatment and the corresponding temperatures on those days). Samples were collected over a 3-day period for each timepoint, for logistical reasons.

#### 2.2.1 Gill sampling

On each sampling day, five mussels from each family (A, B, C) and temperature treatment (CTL, RR, RH) were weighed and measured. Each mussel was then opened, and a section of gill was dissected and placed in a 2 mL cryovial before snap-freezing in liquid nitrogen. Gill samples were stored in a −80°C freezer for later analysis (Figure 1B).

#### 2.2.2 Hemolymph extraction

For the heat-stress treatments (RH and RR), two mussels from each family were sampled from each tank on each sampling day (e.g., on experimental day 7, 2 mussels from each family were sampled from each “ramp and hold” tank (eight tanks = 16 mussels per family total). For control treatments, there were only two tanks, but the same number of mussels (i.e., eight mussels per family from each tank) were sampled (Figure 1B). Repeated measures were used, so the same mussels were sampled at each timepoint. Significant mortality within the RH treatment by T-3 meant that a reduced number of mussels were available to sample at this time point. Sample sizes for hemolymph extraction at each timepoint are show in Supplementary Table S1. Mussels were removed from their tanks, weighed and measured using calipers. One milliliter of hemolymph was drawn from the mussels’ posterior adductor sinus using a pre-chilled 25G needle equipped with a 1 mL syringe and checked for purity using a compound microscope. Five hundred µL was aliquoted into a pre-chilled 1.5 mL microcentrifuge tube and held on ice. Two-hundred and fifty µL of this was further aliquoted into an equal volume of ice-cold autoclaved filtered seawater (AFSW) to make a “working hemolymph stock” (WHS). The undiluted tube was set aside for total antioxidant capacity (TAC) measurements, while the diluted tube was allocated for flow cytometry. The remaining 500 µL of hemolymph was transferred to a screw-top micro-centrifuge tube and held on ice before centrifuging (3°C, 2500 RPM, 2 min). Supernatant (plasma) was then removed from the pellet to retain hemocytes only, then each vial was snap frozen in liquid nitrogen and stored at −80°C for later transcriptomics analyses (Figure 1B).

### 2.3 Flow cytometry, antioxidant capacity, transcriptomics and microbiome analyses

#### 2.3.1 Hemocyte concentration and viability assay

Fifty microliters of WHS were added to 150 µL of AFSW in lightsafe microcentrifuge tubes (final 8x hemolymph dilution) before 1 µL of concentrated Muse^®^ Count and Viability Kit (200X, part number MCH100104; Luminex) was added. The Muse^®^ Count and Viability dye differentially stains non-viable and viable hemocytes, based on their permeability to the dye reagents. Samples were vortexed for 3 s at 1,400 rpm and incubated at room temperature (17°C) for 5 min before analysis using a Muse^®^ Cell Analyzer (Luminex). Positive controls of dead samples were run by incubating a microcentrifuge tube of hemolymph in a beaker of boiled water for 20 min (Rolton and Ragg, 2020), before running the sample on the Muse^®^ Cell Analyzer. Hemocyte concentration are expressed as cells/mL and non-viable (dead) hemocytes are expressed as a percentage of the total hemocyte concentration.

#### 2.3.2 Hemolymph oxidative stress and antioxidant capacity assays

Oxidative stress in mussel hemocytes was detected using the Muse^®^ Oxidative Stress Kit (part number MCH100111 Luminex). This kit measures the percentage of cells in a sample that have superoxide radicals detected in them (the presence of superoxide is detected using cell-permeable dihydroethidium dye which reacts with superoxide anions). Percentages of superoxide positive hemocytes are expressed as SO+ hemocytes (%).

Fifty microliters of WHS were added to lightsafe microcentrifuge tubes containing 150 µL of Muse^®^ Oxidative Stress reagent (final 8x hemolymph dilution), made up with AFSW instead of the manufacturers assay buffer, to ensure that the osmolality of the mixture was optimized for shellfish hemocytes. Samples were vortexed for 3 s at 1,400 rpm and incubated at room temperature (17°C) for 30 min before analysis on the Muse^®^ Cell Analyzer. Positive control reactive oxygen species (ROS) samples were run by adding menadione at a final concentration of 0.10 mM to the concentrated hemolymph sample, and incubating on ice for 20 min, before running on the Muse^®^ Cell Analyzer as above as a regular sample. Total antioxidant capacity (TAC) was measured in 70 µL of hemolymph which were aliquoted onto a disposable strip and analyzed electrochemically using a portable total antioxidant capacity device (e-BQC, Bioquochem S.L.) and expressed in microcoulomb (µC) (Delorme et al., 2021). The e-BQC device estimates the antioxidant components of a biological sample and its potential to scavenge free radicals. This can provide a measure of physiological stress in an organism, as observed in other recent studies (Delorme et al., 2021; Lulijwa et al., 2021; Tuckey et al., 2023).

#### 2.3.3 Transcriptomics (hemolymph)

RNA was extracted from hemocyte samples using the RNeasy Mini Kit (Qiagen) following the manufacturer’s protocol. Residual genomic DNA was removed by treating the total RNA with Turbo DNAse (Invitrogen, Auckland, NZ), following the manufacturer’s protocol. RNA concentration and purity were measured using a NanoPhotometer^®^ N60/N50 (Implen GmbH, Munich, Germany). The 119 samples were sent to Azenta Life Sciences (Suzhou, China) using the provided RNA Stabilization tubes for sample QC, library preparation (Poly A enrichment, non-strand specific) and Illumina NovaSeq sequencing (150 bp paired-end reads, minimum 10 M reads per sample). Purified total RNA (2–20 μg) was pipetted into RNA Stabilization tubes for analysis. Raw sequence data were processed with the Oyster River Protocol (ORP; version 2.3.3; Macmanes, 2018), a benchmarked bioinformatics program specialized in *de novo* transcriptome assembly and analysis. First, Illumina sequencing adapters and nucleotides with low Phred score ( ≤ 2) were removed using Trimmomatic (version 0.39; Bolger et al., 2014). Sequences were then quality filtered and error corrected with the Rcorrector program (Song and Florea, 2015). Due to the large number of samples and size of the data, a subset of samples (one sample per treatment, family and sampling point) were selected to create the assembly. Representative samples were selected based on the purity (260/280 and 260/230 ratio) and concentration of the RNA extracts, the quality score attributed by the sequencing facility, and the number of sequenced reads. A subset of samples from all temperature treatments were chosen for the *de novo* assembly, for a total of nine samples (see Supplementary Table S3).

A total of three assemblies were created by the ORP protocol, two with Spades (kmer of 55 and 75; Bankevich et al., 2012) and one with TransABySS (kmer of 32; Robertson et al., 2010). These assemblies were subsequently merged with the OrthoFuse program (Macmanes, 2018), and quality assessed using both TransRate (version 1.0.3; Smith-Unna et al., 2016) and the Benchmarking sets of universal Single-Copy Orthologs (BUSCO; version 5.1.2; Seppey et al., 2019) against the mollusca_od10 reference database. Sequences were annotated with the EnTap program (version 0.10.8; Hart et al., 2020) using the EggNOG (Huerta-Cepas et al., 2018), UniRef90 (Suzek et al., 2014), RefSeq (O'Leary et al., 2015), and the UniProt (includes Swiss-Prot and TrEMBL databases; Bateman et al., 2021) databases. Sequences from all samples were mapped to the final *de novo* assembly, and quantified with the SALMON program (version 1.4; Patro et al., 2017).

#### 2.3.4 Microbiome (gill)

DNA was extracted from gill samples using a DNeasy PowerSoil Pro DNA extraction kit (Qiagen, Hilden, Germany) following the manufacturer’s protocol. The bead-beating step for the Power Soil extraction was carried out on a 1,600 MiniG automated tissue homogenizer (SPEX Sample Prep, Metuchen, United States) at 1,500 rpm for 2 minutes.

Polymerase chain reaction (PCR) was carried out on all samples including blank controls using the universal bacterial primers S-D-Bact-0341-b-S-17 (forward): 5′-CCT ACG GGN GGC WGC AG-3′ and S-D-Bact-0785-a-A-21 (reverse): 5′-GAC TAC HVG GGT ATC TAA TCC-3′ targeting regions V3 and V4 of the 16S rRNA gene (Klindworth et al., 2012). The universal 16S rRNA gene primers were modified to include Illumina™ overhang adaptors (forward: 5′-TCG TCG GCA GCG TCA GAT GTG TAT AAG AGA CAG-3′ and reverse: 5′- GTC TCG TGG GCT CGG AGA TGT GTA TAA GAG ACA G-3′), as described in Kozich et al. (2013). PCR of bacterial 16S rRNA gene sequences were carried out in 50 μL reaction volumes containing 25 μL MyFi 2× PCR Supermix (Bioline, London, UK), 0.20 μM of modified Illumina overhang adaptor primers, 4 μL of template DNA. Polymerase chain reaction thermocycling conditions were: 94°C for 3 min, followed by 30 cycles of 94°C for 30 s, 52°C for 30 s, 72°C for 1 min, with a final extension step at 72°C for 5 min.

Amplicon libraries were purified with AMPure^®^ XP PCR Purification beads (Agencourt^®^, MA, United States) following the manufacturer’s instructions, quantified using a Qubit^®^ Fluorometer (Life Technologies, Carlsbad, CA, United States), and diluted to 3 ng/μL with ddH_2_O. Purified amplicon libraries were individually indexed using the Nextera™ DNA library Prep Kit (Illumina, California, United States) through an eight cycles PCR, and sequenced on a MiSeq Illumina™ sequencer by a commercial provider (Sequench, Nelson, New Zealand).

Sequenced data were demultiplexed and primers removed using CUTADAPT (version 3.7; Martin, 2011), requiring a minimum overlap of 17 bp and no insertion or deletion. Forward and reverse reads were truncated at 226 and 220 bp on their 3′ end, respectively, to remove low-quality bp, and quality filtered and denoised using the default parameters of the DADA2 R package [version 1.21; Callahan et al. (2016)]. Reads were merged using a minimum overlap of 10 bp, and potential chimeric sequences removed using the ‘consensus’ option of DADA2. The remaining sequences were taxonomically assigned using the SILVA reference database (version 138 clustered at 99% similarity; Quast et al. (2013) using DADA2’s inbuilt RDP Naïve Bayesian Classifier (Wang et al., 2007). Sequences found in blanks were investigated and potential contamination removed using the microDecon R package [version 1.0.2; McKnight et al. (2019)] using the default settings. Sequences unclassified at kingdom level or not identified as bacteria were discarded. Sequencing depth and diversity coverage per sample were investigated with rarefaction curves using the “ggrare” function of the ranacapa R package [version 0.1.0; Kandlikar et al. (2018)] and samples with less than 1,000 reads were removed from downstream analysis.

### 2.4 Statistical analyses

#### 2.4.1 Survival

Survival data were analysed in RStudio (v 1.4.1717). Separate Kaplan-Meier analyses were run with family and treatment as factors and a family*treatment interaction, and a log-rank X^2^ test statistic was obtained for each analysis. Pairwise comparisons using the log-rank test were carried out to investigate differences between factor levels.

#### 2.4.2 Hemolymph flow cytometry and antioxidant capacity statistics

Statistical analyses were undertaken in Rstudio (v 1.4.1717). Flow cytometry data [hemocyte concentration (cells/mL), dead hemocytes (%), superoxide positive hemocytes (%)] and TAC data (total antioxidant capacity (Qt µC)) were analyzed using a linear mixed effects model using the lme4 package.

Treatment (three levels: control (CTL), ramp and recover (RR), ramp and hold (RH)), Family (three levels: A, B, C), Time (timepoints T-1, T-2, and T-3) and their interactions were included as fixed effects in the model. Subjects (individual mussels) were included in the model as a random factor to account for the repeated measurements of the hemolymph (e.g., as the samples from the individuals were not independent). Tank was also included as a random factor, with subjects nested within tanks. Three-way interactions (Treatment*Family*Time) were dropped from models when they were not statistically significant and AIC levels suggested that model simplification was preferable.

Histograms and residual vs. fitted values plots were visualized to ensure that data met assumptions of normality and homogeneity of variances, respectively. Data that did not meet these assumptions were either log transformed (dead hemocytes) or square-root transformed (SO+ cells and Q1) to meet these assumptions. The lsmeans package was used to conduct Tukey *post hoc* tests (with alpha = 0.05) on significant main effects and interactions, to investigate differences between factor levels.

#### 2.4.3 Transcriptomics

Quantified and annotated transcriptomic data was imported into Rstudio (R Core Team 2020) using the tximport package (Soneson et al., 2016), rare transcript and genes (those with 10 reads or less within 3 or less samples) were removed, and the remainder transformed using the vst function of DESeq2 package (Love et al., 2014) to stabilize variance. Principal Component Analysis (PCA) based on Euclidean dissimilarity using the vegan package (Oksanen et al., 2020) and ggplot2 packages (Wickham et al., 2016) was applied to visualize the differences between treatments and families per sampling point. The effect of treatment, family and their interaction per sampling point on the whole transcriptome, both at transcript and gene levels, was evaluated with a permutational analysis of variance (PERMANOVA) using the adonis function of the vegan R package with 999 permutations. To give an idea of how the number of differentially expressed transcripts (DET) and genes (DEGs) changes per treatment through time, data between sampling point 1 with sampling point 2 and 3 were compared, and number of DEGs visualized with barplots using ggplot2, and Venn diagrams using the eulerr R package (version 7.0.0; Larsson (2022). The top 20 DEGs with lowest p.value per treatment and family at sampling point 3 were further visualized with heatmaps using the ComplexHeatmap R package (version 2.14.0; Gu (2022). Significantly enriched pathways (herein GO terms) were identified using the gene set enrichment analysis approach (GSEA, Subramanian et al., 2005) with gene level data and the fgseaMultilevel function of the fgsea R package (Korotkevich et al., 2016), and the 10 pathways with lowest *p*.values per treatment and ontology (molecular function vs. biological process vs. cellular component) were visualized with ridgeline plots using the ggridges R package [version 0.5.4; Wilke (2022)].

#### 2.4.4 Metabarcoding statistics methods

The microbial taxonomic composition was visualized with barplots using the ggplot2 (version 3.3.6; Wickham et al., 2016) and biohelper R packages (version 0.0.8, Laroche et al., 2018).

Alpha-diversity metrics, herein observed richness and Shannon diversity, were assessed on rarefied data (9,850 reads/sample) with the phyloseq R package (version 1.40.0; Callahan, et al., 2016) and visualized with boxplots. Differences between families, treatment and sampling point were investigated with independent samples *t*-Tests whenever the fourth root transformed values respected the normality assumption (tested with Shapiro-Wilk test) and equality of variance (tested with Levene’s test). In case one of these assumptions was violated, an independent samples Wilcoxon test was performed. All tests were conducted with the rstatix R package (version 0.7.0; Kassambara, 2021).

Beta-diversity was visualized with Principal Components Analysis (PCA) and the effect of family, treatment and their interaction tested per timepoint with a permutational analysis of variance (PERMANOVA; permutations = 999, by = “terms”) and the “adonis2” function of the vegan R package (version 2.6.2; Oksanen et al., 2020). These analyses were performed on rarefied data (9,850 reads/sample) transformed to relative abundance and the Bray-Curtis distance method.

Taxa (species and genera) differentially abundant between treatment at the end of the experiment were identified with the ANCOMBC R package (version 1.6.2; Li et al., 2020) using default parameters, and visualized with barplots. In addition, putative pathogen taxa were identified and highlighted by looking at 100% similarity matches with sequences from the prokaryote pathogen database created by Yang et al. (2023).

## 3 Results

### 3.1 Seawater temperature and dissolved oxygen

Seawater temperatures (°C) and dissolved oxygen concentrations (mg/L) (mean ± SD) for each temperature treatment (CTL, RR and RH) during the 36-day experiment are shown in Figure 2.

### 3.2 Survival

**FIGURE 3:**
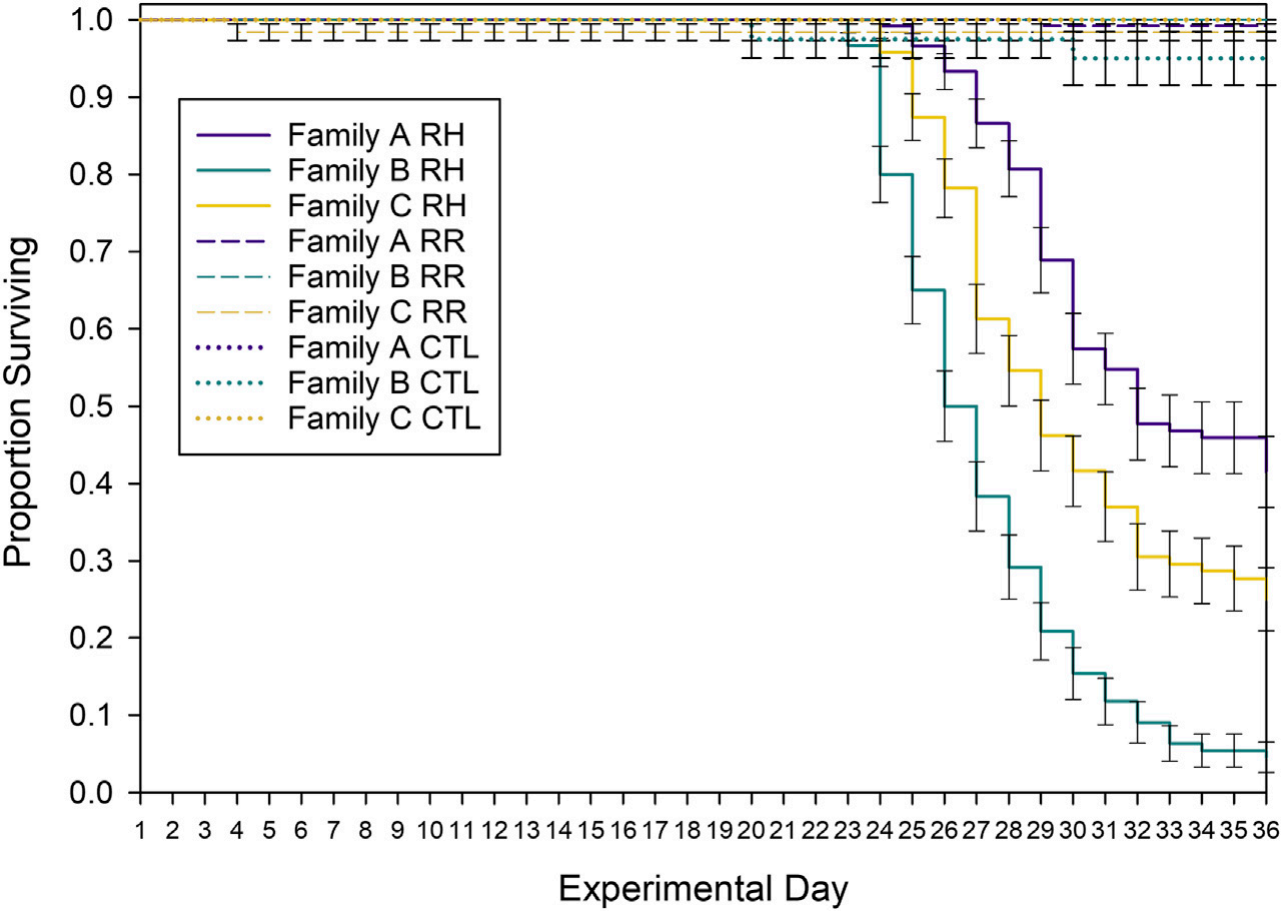
Kaplan Meier survival curves for each experimental treatment. Control (CTL), ramp and hold (RH) and ramp and recover (RR) treatments and family (A, B, C) are shown over the 36-day experimental trial. Error bars denote standard error.

Mussel survival differed between families (Family Log-rank X^2^ = 22.9; *p* < 0.001) and temperature treatments (Treatment Log-rank X^2^ = 570; *p* < 0.001) and an interaction between family and treatment indicated that family differences depended on treatment (Family * Treatment Log-rank X^2^ = 770; *p* < 0.001). Survival in the CTL and RR treatments was high (>90%) and did not differ (*p* = 0.410), but survival was lower in the RH treatment, compared with other treatments (*p* < 0.001). Within the RH treatment, the survival of all families was significantly different to one another (*p* < 0.001; Supplementary Table S2). Family A had the highest survival rate by day 36 (42%), followed by family C (25%) and family B (5%) (Figure 3).

Mussels began to die in the RH treatment once the seawater temperature had been at 26°C for 5 days. Because the mussels are the same age but have a range of shell lengths (e.g., Family A is much smaller than families B and C), it is important to examine the relationship between mussel size and heat susceptibility. When the shell length of mortalities is plotted against the number of days held at 26°C, there was no relationship between mussel size and ‘days to death’ aside from a weak trend for family B (Supplementary Figure S1, *R* ^2^ = 0.092). This indicates that mussel size within each family was not correlated with susceptibility to heat-stress.

### 3.3 Immune response (hemolymph)

**FIGURE 4:**
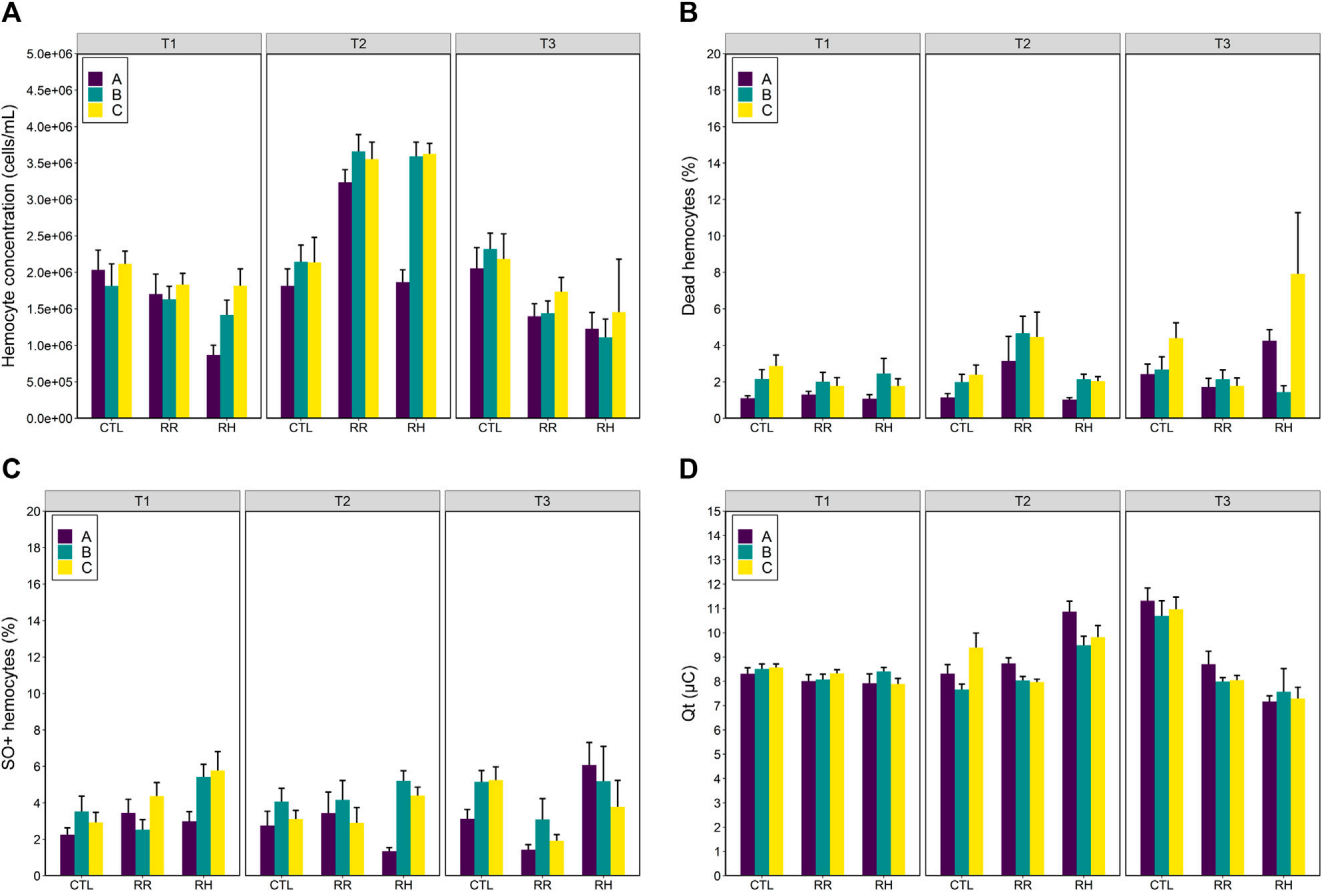
Hemolymph immune responses **(A)** hemocyte concentration (cells/mL); **(B)** dead hemocytes (%); **(C)** SO+ hemocytes (%) and **(D)** total antioxidant capacity (Qt µC) of selectively bred *Perna canaliculus* to three different seawater trajectories (control (CTL), ramp and recover (RR) and ramp and hold (RH)). Responses are shown for three mussel families (A, B and C). Error bars denote standard error.

Hemocyte concentration increased in mussels exposed to heat-stress, as observed at sampling timepoint T2 (Figure 4A). Hemocyte concentration did not differ between timepoints for mussels in the control treatment (Treatment*Time *p* > 0.001; Tukey *p* > 0.5), but hemocyte concentration were higher at T2 in mussels from the RR and RH treatments compared with the other timepoints (Tukey *p* < 0.001).

Family A differed in its hemocyte concentration compared with the other families when exposed to heat stress. Family A had lower hemocyte concentration than family C, but only in the RH treatment (Treatment*Family *p* < 0.002; Tukey *p* < 0.001). Family A had lower hemocyte concentration than families B and C at timepoint 2 (Family*Time *p* < 0.003; Tukey *p* < 0.001), but not at the other timepoints (Tukey *p* > 0.10).

Percentages of dead and SO+ hemocytes were less influenced by heat stress compared with hemocyte count (Figures 4B, C). Family A had lower percentages of dead hemocytes than family C, but this was not correlated with heat-stress as it only occurred in the control treatment (Treatment*Family *p* < 0.02; Tukey *p* < 0.001). Family A had lower percentages of dead hemocytes than families B and C at timepoint T1 (Family*Time *p* < 0.03; Tukey *p* < 0.04), and at T2 (Tukey *p* < 0.001). There were no differences between the families at T3 (Tukey *p* > 0.08). Percentages of dead hemocytes did not differ between mussels in the different temperature treatments at T1 (Treatment*Time *p* < 0.001; Tukey *p* > 0.693) but were higher in mussels in the RR treatment compared with the RH treatment at T2 (Tukey *p* < 0.03) and were lower in mussels in the RR treatment at T3 compared with the other temperature treatments (Tukey *p* < 0.04).

Similarly, family A had lower percentages of SO+ hemocytes than family C but this occurred in a range treatment and timepoint combinations, and in the control treatment (Treatment*Family*Time *p* < 0.005; Figure 3C). At sampling timepoints T1 and T3, percentages of SO+ hemocytes in mussels were similar to each other (Tukey *p* > 0.196), aside from slightly decreased percentages in family A compared with family C in the control treatment (Tukey *p* < 0.05). At T2, family A had significantly less SO+ hemocytes than family C in the RH temperature treatment (Tukey *p* < 0.001).

The total antioxidant capacity (Qt) of mussels was influenced by seawater temperature (Temperature*Time *p* < 0.001) (Figure 4D). At timepoint T1, levels of Qt did not differ between temperature treatments (Tukey *p* > 0.518). At T2, levels of Qt were higher in mussels in the RH treatment compared to the other treatments (Tukey *p* < 0.001). At T3, mussels in the control treatment had higher levels of Qt than mussels in the other treatments (Tukey *p* < 0.001), and RR mussels had slightly higher levels of Qt than RH mussels (Tukey *p* = 0.038). The only difference observed between families was a slightly lower level of Qt in mussels from family B compared with A and C at T2 (Family*Time *p* < 0.004; Tukey *p* < 0.04) (Figure 4D).

### 3.4 Transcriptome

A total of 1,316.5 million (M) reads were generated from the 114 libraries (Supplementary Table S3), for a mean value of 11.5 M per sample. The generated merged assembly comprised 144,788 transcripts for a total size of 129.1 MB with a mean GC content of 33%. BUSCO analysis showed a completeness of 65.3% (56.9% as singletons and 8.4% as doubletons) against the mollusca_od10 core gene database, with 4.7% and 30% of fragmented and missing genes, respectively. The optimal TransRate score was assessed at 0.34, with a n50 of 1,655 base-pair (bp) (Supplementary Table S4). Among all transcripts, 23.8% (34,528) showed evidence of containing open reading frames (ORF). Among these ORF sequences, 76.9% (26,547) could be annotated, 57.7% (19,923) assigned to a gene ontology (GO) term, and 42.9% (14,826) could be assigned to a gene. The mean read mapping rate against the assembly was 94.5% (Supplementary Table S3). Due to having relatively low mapping rate (≤ 86%), two samples (GSM-213 and GSM-396) were discarded. Removing rare transcripts in samples led to a total of 49,116 unique transcripts.

**FIGURE 5:**
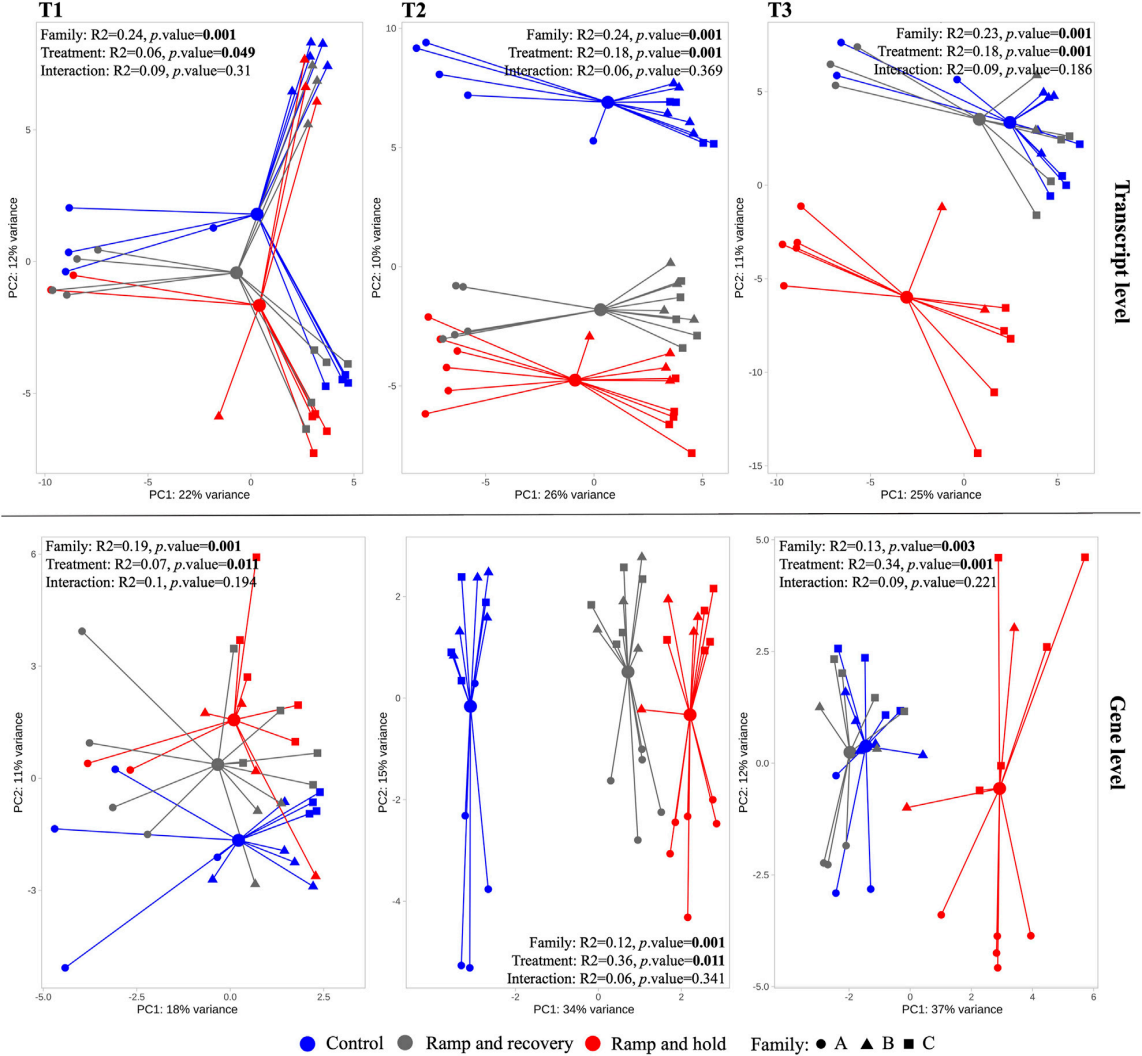
Principal Component Analysis (PCA) of transcriptomes per sampling timepoint (T1-T3) at transcript and gene levels. The larger circles indicate centroid per treatment, linked with colored segment to associated samples. Results of the permutational analysis of variance (PERMANOVA) testing the effect of family, treatment and their interaction effect is displayed within each facet, with significant effects highlighted in bold.

Figure 5 shows ordinations and PERMANOVA results per sampling timepoint (T1 [before temperature treatment], T2 and T3). There is no interaction effect between family and temperature. The effect of temperature treatment at timepoint T1 is significant (albeit marginal) and increases substantially at T2 and T3 as can be observed in the PCAs and PERMANOVA results. The pairwise PERMANOVA shows that at T3 RR samples are no longer significantly different to that of controls (Table 1). Of note is that although pairwise PERMANOVA results show no significant differences between families, Family A generally appears more dissimilar to B and C at all sampling points, and especially in its response to RR and RH treatments at T3 according to the R^2^ pairwise PERMANOVA results in Table 1.

**TABLE 1:** Pairwise permutational analysis of variance testing all pairs of treatments per sampling time point and pairs of families per treatment at sampling point 3, at transcript level. Significant *p* values are highlighted in bold. RH = Ramp and Hold; RR = Ramp and Recovery; CTL = Control.

Sampling time point	Pairs	*R* ^2^	*p*. value adjusted	Treatment	Pairs	*R* ^2^	*p*. value adjusted
T-1	RH vs. RR	0.10	0.076	Ctrl	A vs. B	0.17	0.876
	RH vs. CTL	0.25	**0.003**		A vs. C	0.17	1
	RR vs. CTL	0.13	**0.012**		B vs. C	0.05	1
T-2	RH vs. RR	0.13	**0.003**	RR	A vs. B	0.21	1
	RH vs. CTL	0.49	**0.003**		A vs. C	0.43	0.087
	RR vs. CTL	0.37	**0.003**		B vs. C	0.29	0.6
T-3	RH vs. RR	0.36	**0.003**	RH	A vs. B	0.23	0.133
	RH vs. CTL	0.28	**0.003**		A vs. C	0.20	0.057
	RR vs. CTL	0.10	0.165		B vs. C	0.14	1

**FIGURE 6:**
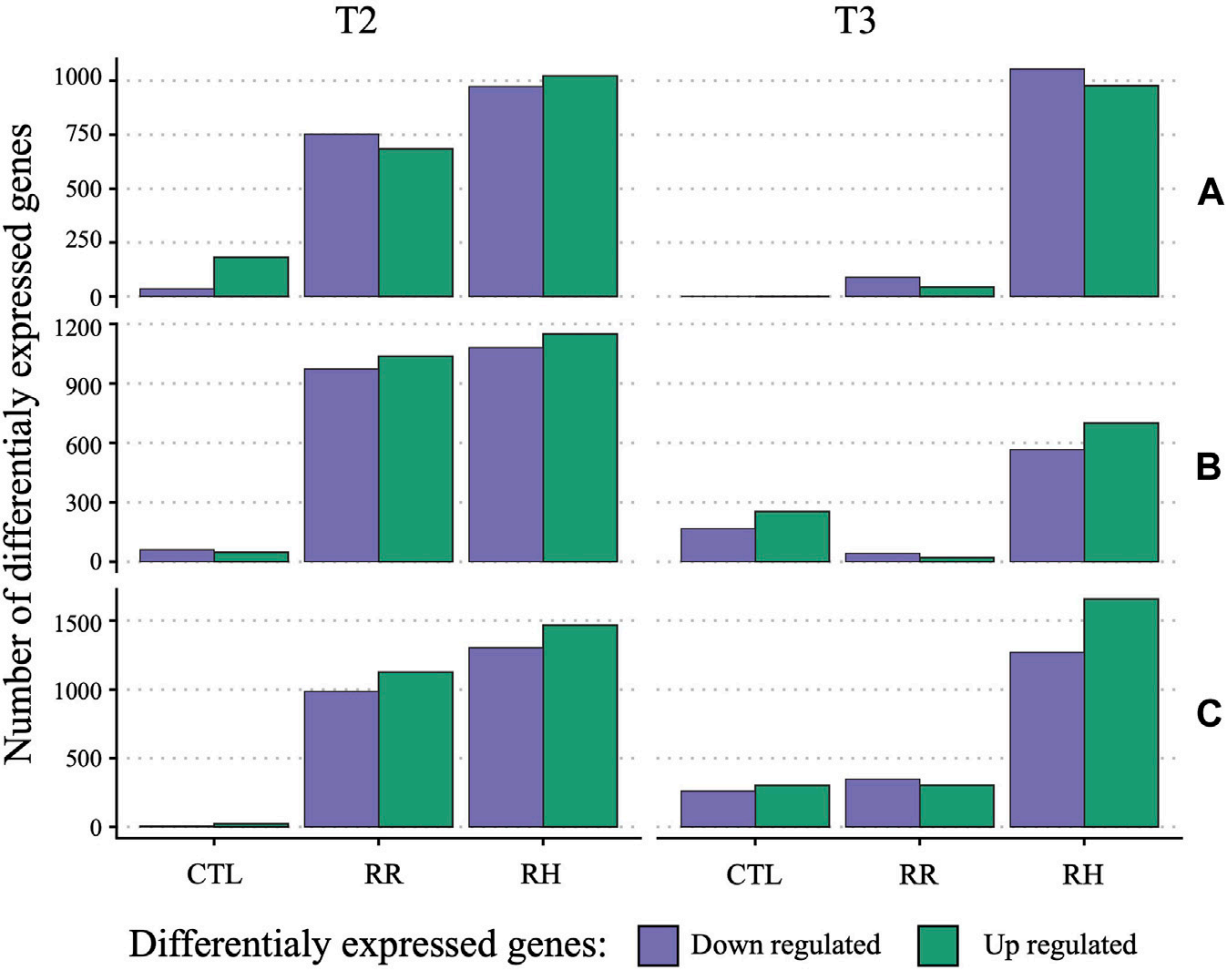
Barplot of differentially expressed genes per family (A, B and C) and sampling timepoints (T2 and T3) against respective controls at T1. RH = Ramp and Hold; RR = Ramp and Recovery; CTL = Control.

Genes that were differentially expressed (DEG) due to treatment and sampling timepoint were identified using DeSeq2 per family against controls at timepoint T1 (Figure 6). For controls, few genes were differentially expressed through time, especially between T1 and T3. At T2, the number of DEG was relatively similar between RH and RR. This changes at T3, where the number of DEG return closer to control levels for RR samples. Interestingly, family C had substantially more genes impacted by treatment and time point than the A and especially B family (2769 [RH] and 2112 [RR] for family C *versus* 2232 [RH] and 2012 [RR] for family B at T2, and 2925 [RH], 650 [RR] *versus* 1,267 [RH] and 62 [RR] for family C and B, respectively, at T3).

**FIGURE 7:**
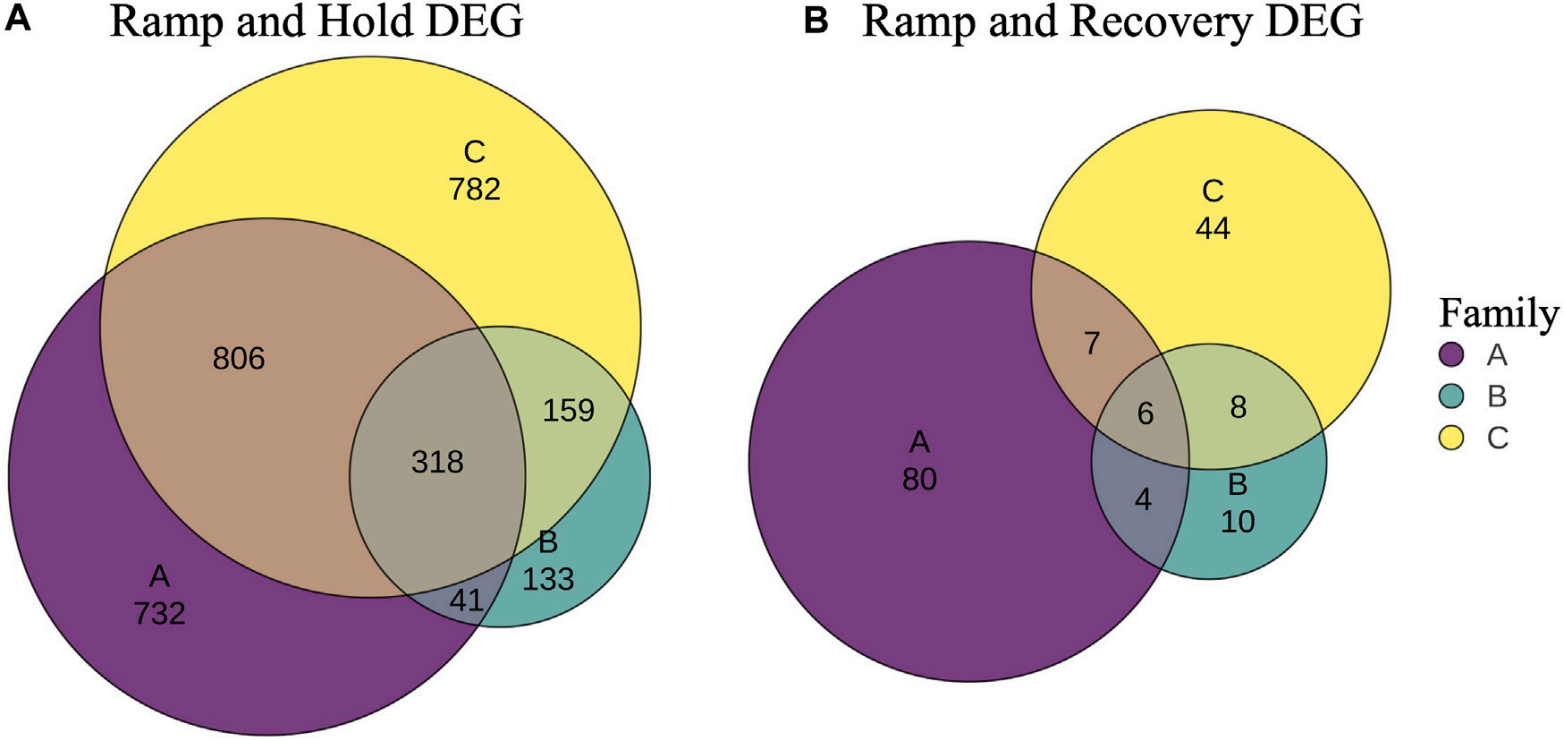
Venn diagrams of unique and shared differentially expressed genes (DEG) between controls and ramp and hold **(A)** and between controls and ramp and recover samples **(B)** at sampling time point 3 (T3).

In addition, genes that were differentially expressed due to treatment at sampling timepoint T3 were identified per family with DeSeq2 (Supplementary Table S5). In general, slightly less than half of the DEGs between controls and RH were unique to each family (Figure 7), with 14% (318) shared across all families. The proportion of family specific DEGs increased drastically between controls and the RR treatment, with less 3% (6) shared across families (Figure 7).

**FIGURE 8:**
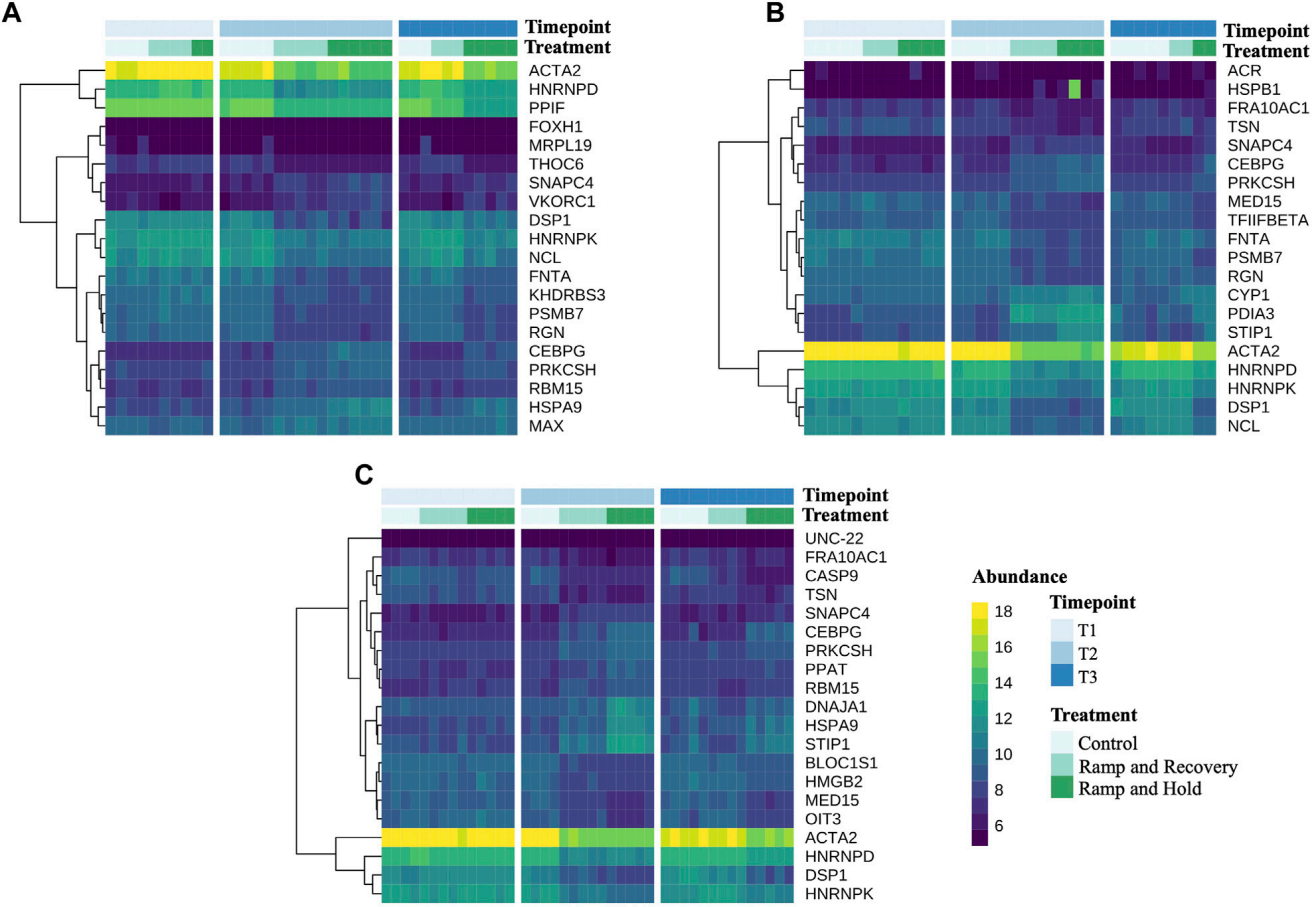
Heatmaps of count data of the 20 differentially expressed genes with the lowest *p* values between control and heat stress treatments at sampling timepoint 3 per family. Supplementary Table S6.

Figure 8 highlights the 20 DEGs between controls and treatments at both timepoints T2 and T3 with lowest *p* values, per family (see Supplementary Table S6 for full gene names listed next to their abbreviations). It notably shows that a small cluster of genes that are highly expressed in control mussels are downregulated by mussels in the increased water temperature treatments, including ACTA2, HNRNPD, HNRNPK, NCL and DSP1. Genes that were upregulated by the treatment were more variable between families and included CEBPG, PRKCSH, RBM15, HSPA9 and MAX for family A, CYP1, PDIA3, STIP1 for family B, DNAJA1, HSPA9 and STIP1 for family C.

DEGs that were common to all families from the RH treatment (318 genes; Figure 7) were further investigated to determine if any showed potential as heat stress biomarkers. DEGs that have identified as having roles as molecular chaperones, related to immune/stress response, temperature homeostasis, and apoptosis were selected to look for consistent response over all families and treatments (Supplementary Figure S2). For examples, some DEGs were upregulated during heat treatments, sustained for the period of the experiment, and then returned to base levels once temperatures were reduced (e.g., AIF1, CTSC, TOLL8–immune response; ARRDC3–temperature homeostasis; ATP6 genes, MYC–apoptosis; GRPEL1, HSP90B1, HSPE1, PPIB–protein folding/chaperone; CRYAB, SNX9, PTGS2–response to stress/ROS; Supplementary Figure S2). In contrast, some DEGs were downregulated in response to increased temperature and then were upregulated again once temperature returned to ambient (e.g., DSP1, FCER2, PIK3R4–immune response; CASP9, FAM genes, FNTA–apoptosis; PFDN4, PPD3–protein folding/chaperone; AHCY, PPIF–metabolism and oxidative stress response).

**FIGURE 9:**
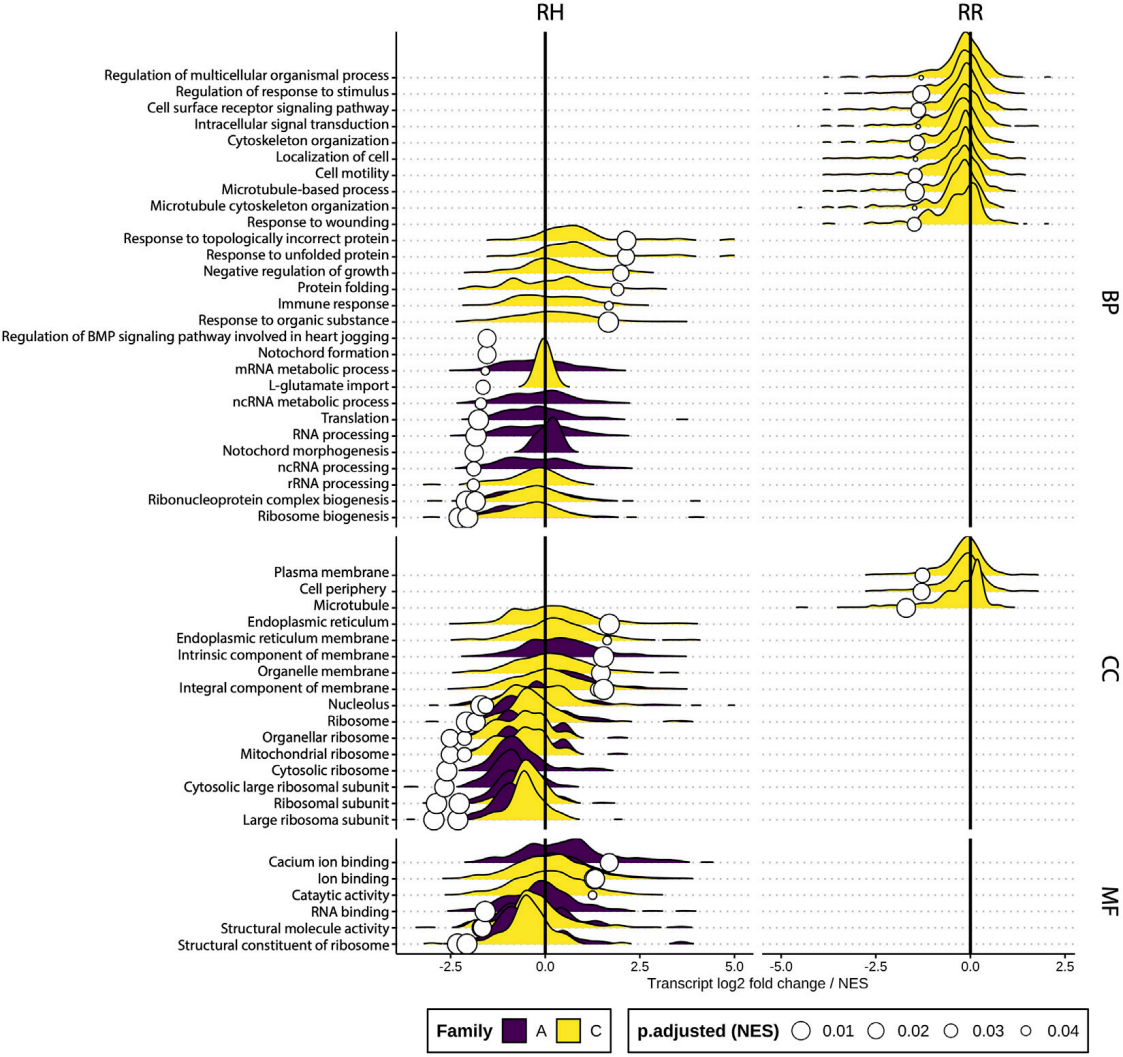
Ridge plots of significantly differentially enriched gene sets per ontology category and treatment at sampling timepoint T3. The density gives an indication of the number of genes with similar log2 fold change per GO term along the *X*-axis (truncated at −5 and 5) while the white circles represent the normalized enrichment score (NES). RH = Ramp and Hold, RR = Ramp and Recover, BP = Biological process, CC = Cellular component, MF = Molecular function.

The effect of sustained heat stress and recovery was examined by performing a differential gene set enrichment analysis (GGSEA) at different gene ontology (GO) levels by contrasting gene abundance between controls and heat stress treatments at sampling timepoint T3 (Supplementary Table S7). Figure 9 shows these results per ontology category (Biological process, Cellular component and Molecular function). Most enriched GO terms belonged to the biological process category. GO terms related to RNA processing were downregulated in the RH treatment, while those relating to protein folding and immune response were upregulated. For the cellular component GO terms, ribosomal GO terms were downregulated and those related to membrane function were upregulated. Upregulated molecular function GO terms included ion binding and catalytic activity, while structural molecule GO terms were downregulated. Overall, Family C had the highest number of differentially expressed GO terms, followed by family A, while family B had none for either treatment. Interestingly, the figure indicates that after recovery, Family C remained more impacted than the other families, with several GO terms being suppressed compared to controls, while families A and B had no affected GO terms after recovery. Figure 8 also highlights that GO terms affected during the sustained increase in water temperature were not necessarily the same impacted during recovery.

### 3.5 Gill microbiome

**FIGURE 10:**
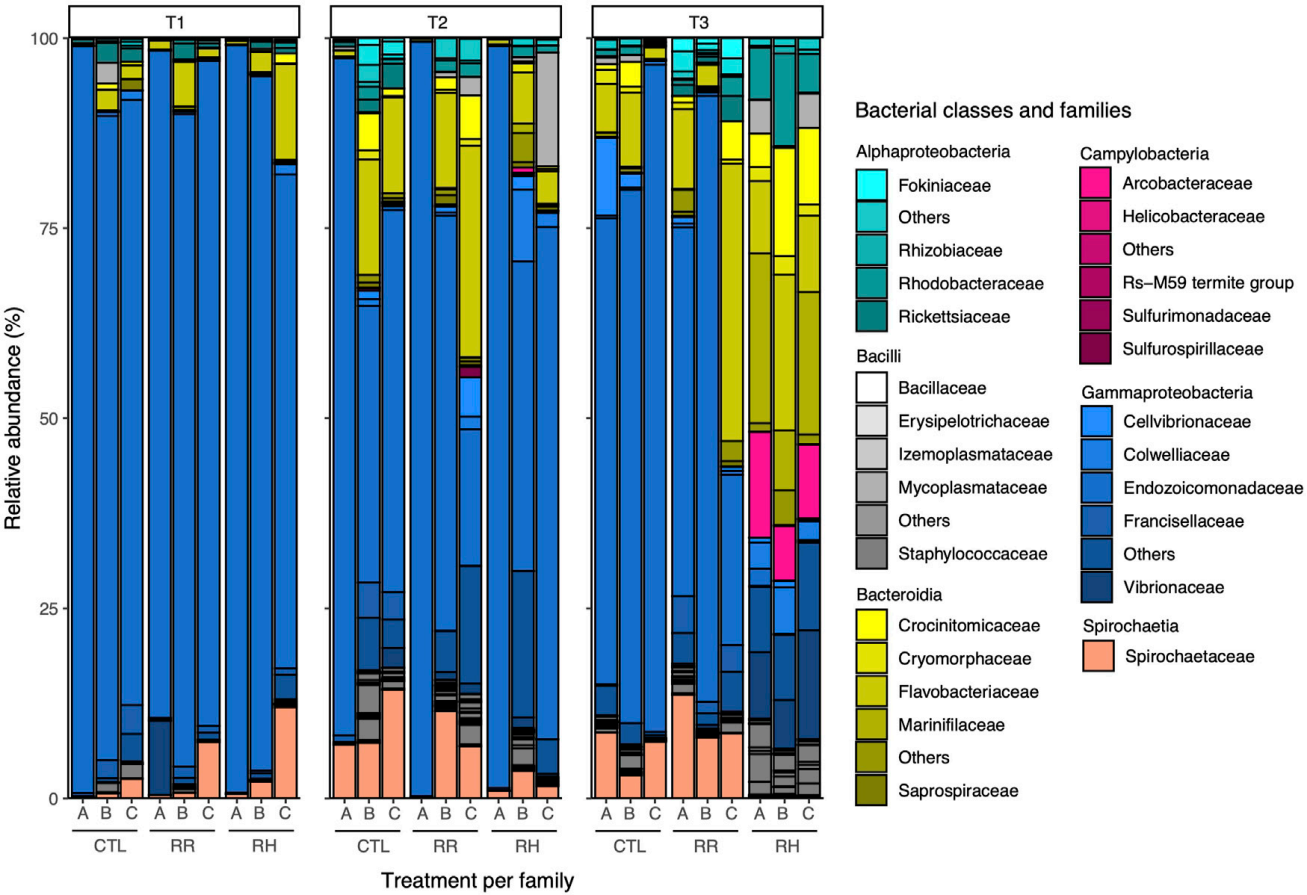
Relative abundance of green-lipped mussel microbial taxa at class and family level per sampling point, family and treatment. CTL = Control, RR = Ramp and recover, RH = Ramp and hold, SP = Sampling point. CTL = Control, RR = Ramp and recover, RH = Ramp and hold, T = Timepoint.

Figure 10 displays the taxonomic composition of the green-lipped mussel microbiome per sampling timepoint, treatments and families at class and family level. It shows a particularly high dominance of Gammaproteobacteria, especially the Endocozoicomonadaceae family, at T1 and within the control samples. Interestingly, the composition changes slightly at T2, including for the control samples, with Spirochaetaceae and Bacteroidia such as Flavobacteriaceae increasing in abundance. Changes in the control mussels likely reflect associated changes within the eutrophic pond communities that are supplying the tanks, as these mussels were not exposed to heat stress. At T3, a drastic difference can be observed between the control and RR samples with those of RH, where the dominance of Gammaproteobacteria has been replaced by Bacteroidia, including Crocinitomicaceae and Flavobacteriaceae in the RH mussels. In addition, *Campylobacter* and the Vibrionaceae family can be found in higher abundance in the RH samples at T3 while the Spirochaetaceae has disappeared. These changes observed at T3 in the RH treatment were similar among families (Figure 10).

**FIGURE 11:**
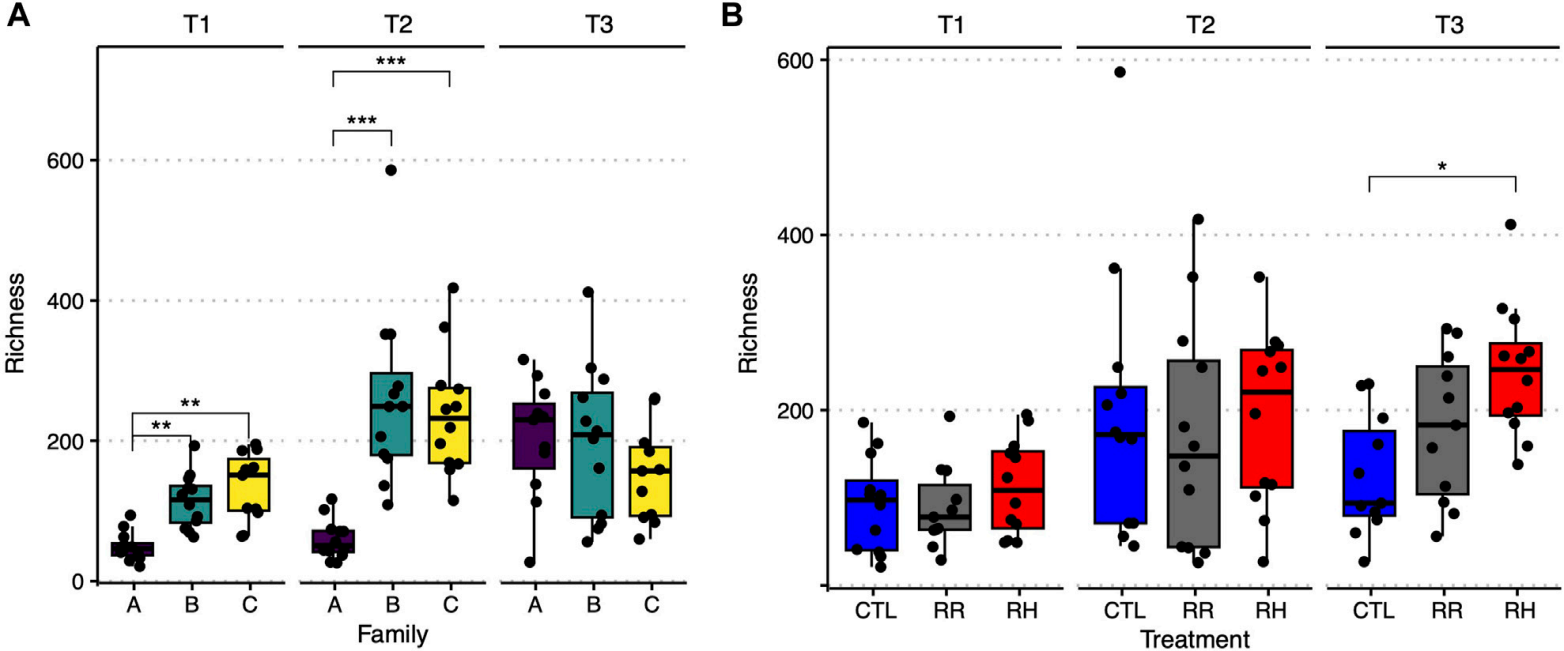
Observed amplicon sequence variant richness per family and sampling timepoint for the control samples **(A)**, and per treatment and sampling point **(B)**. Significant differences between groups are indicated by horizontal lines and stars over the relevant boxplots. CTL = Control, RR = Ramp and recover, RH = Ramp and hold, T1-T3 = Sampling timepoints, * = *p* ≤ 0.05, ** = *p* ≤ 0.01, *** = *p* ≤ 0.001.

Microbial richness in the control samples at the beginning of the experiment was significantly different between family A *versus* families B and C, with ASVs that were at least two times lower in A (Figure 11A). Interestingly, this difference disappeared towards the end of the experiment, with an overall increased richness in all families. Microbial richness was also compared between treatments per sampling timepoint. No difference could be observed until T3, where bacterial richness in the RH mussels was significantly higher than control mussels (Figure 11B). Results for Shannon-diversity were similar to those for richness (see Supplementary Figure S3).

**FIGURE 12:**
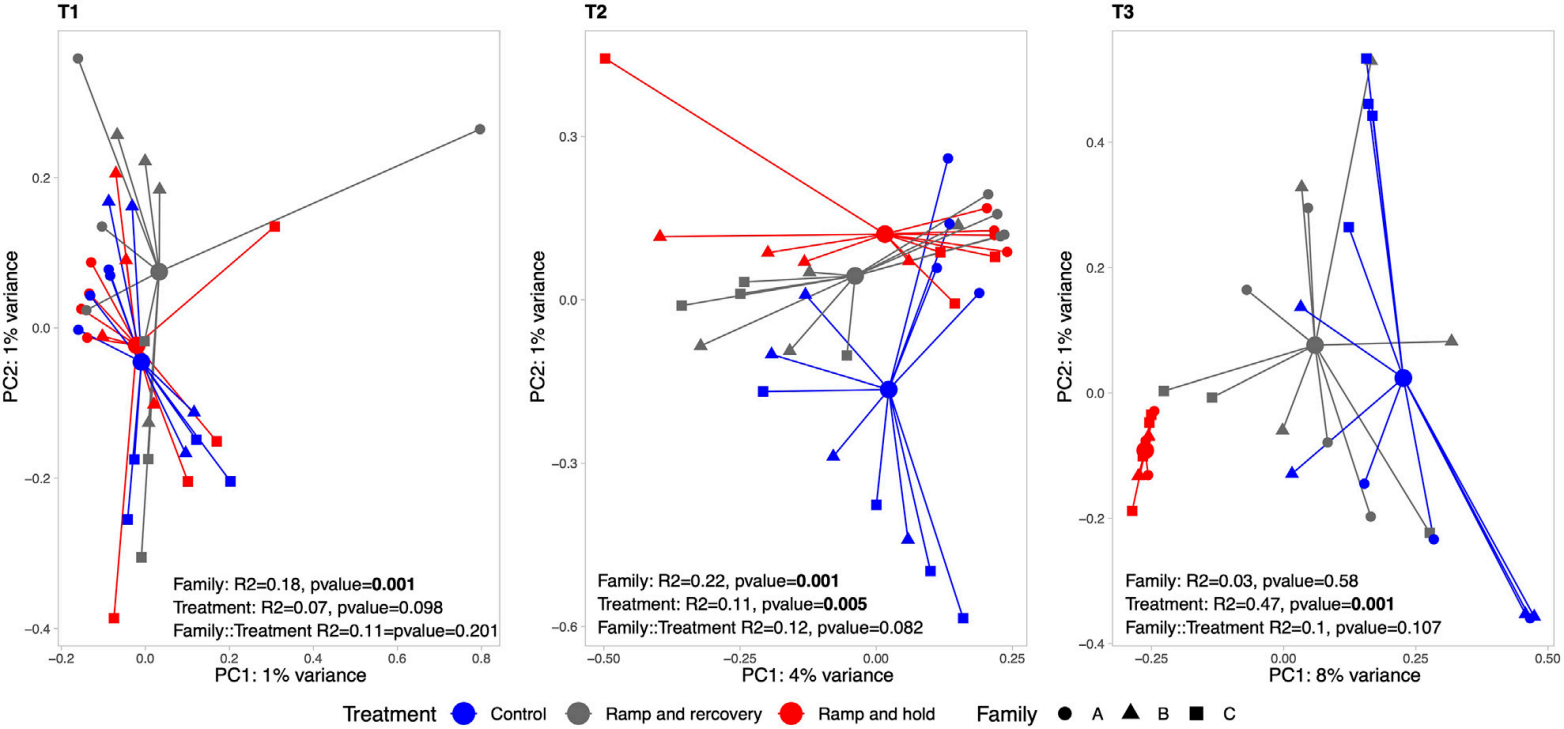
Principal Component Analysis (PCA) of green-lipped mussel microbiome per sampling timepoint (T1, T2 and T3). The larger circles indicate centroid per treatment, linked with colored segment to associated samples. Results of the permutational analysis of variance (PERMANOVA) testing the effect of family, treatment and their interaction effect is displayed within each facet.

Looking at beta-diversity, Figure 12 shows that green-lipped mussel microbiomes were similar between treatments at T1 but started to differ between controls and RR and RH samples at T2. At T3, a strong clustering with reduced variance can be found for RH samples compared to RR and controls. Interestingly, the microbiome of the green-lipped mussels significantly differed between families (*R* ^2^ = 0.18, *p* = 0.001) at the beginning of the experiment but this difference became non-significant towards the end (*R* ^2^ = 0.03, *p* = 0.58). No significant interaction could be found between families and treatment.

A total of 1 species and 5 genera were found differentially abundant between controls and RR samples at T3, including *Planktomarina temperate* and *Mycoplasma* more abundant in controls; and [*Caedibacter*] *taeniospiralis* group, *MD3-55* and *Candidatus Megaira*, more abundant in the RR samples (Supplementary Figure S4B). For RH, the number of differentially abundant taxa was over 10 times higher with 69 identified species and genera (Supplementary Figure S4A). Among those, 5 were found more abundant in the control samples, including *Endozoicomonas*, *Lutibacter*, *Fluviicola*, Clade Ia and *Cycloclasticus*. Among taxa affiliated with RH samples, those with highest log fold change included *Malaciobacter*, *Pseidonibacter*, *Catenococcus*, *Salinirepens* and *Tropicibacer phthalicicus*. In addition, a few of these RH associated taxa were identified as putative pathogenic bacteria, including *Vibrio fortis*, *Vibrio tubiashii*/*xuii* (100% similarity to both species) and *Vibrio mediterranei*/*shiloi*.

## 4 Discussion

### 4.1 Survival and immune response

Prolonged exposure to seawater temperatures of 26°C were detrimental to the survival of *P. canaliculus*, with significant mortality beginning after 5 days at this temperature (RH treatment). Selectively bred families differed significantly in their susceptibility to thermal stress, with family A having the highest survival rate at the end of the trial (42%), followed by families C (25%) and B (5%). These results show that genetics play a fundamental role in determining thermal tolerance in *P. canaliculus*, although the heritability of this trait remains to be investigated.

The results observed in this study, along with other recent studies using this 26°C survival challenge method (Ericson et al., 2023a) supports the use of a sustained 26°C heat challenge as an effective method to compare heat tolerance of selectively bred *P. canaliculus*. Survival challenges that monitor mortality during heat stress are used to compare tolerance in other selectively bred aquaculture species including species of oysters (Camara et al., 2017; Ding et al., 2020; Scanes et al., 2020) and trout (Gallardo-Hidalgo et al., 2021). Although this method is effective, the development of non-lethal methods to measure and predict heat-tolerance is preferable, as survival challenges result in mortalities of valuable stock. Development of non-lethal biomarkers for heat tolerance were therefore the primary focus of this study, with a survival challenge included for validation.

The hemolymph immune response of mussels in this study was complex and variable, making it challenging to use this as a robust, consistent stress biomarker. We were also limited by the flow cytometer that was used to measure hemocyte responses, as it cannot distinguish between the complex assemblage of cell types in the hemolymph (e.g., blast-like cells, hyalinocytes and granulocytes; Rolton and Ragg, 2020). We could, therefore, only measure changes in the total hemocyte pool and not the sub-populations of hemocytes.

Total mussel hemocyte concentration did respond dynamically to thermal stress, with increased cell concentration seen in mussels when they reached ∼26°C at timepoint 2 (RH and RR treatments). This increase in hemocyte concentration was the strongest immune response detected in this study, and this has also been observed in other marine shellfish exposed to significant thermal stress (Monari et al., 2007; Rahman et al., 2019). Hemocyte concentration can be highly variable depending on the temperature trajectory that an organism has experienced. Previous studies on *P. canaliculus* have found either no effect of temperature on hemocyte concentration (Delorme et al., 2021), or a decrease in hemocyte concentration at elevated seawater temperatures (Ericson et al., 2022; Ericson et al., 2023b). Hemocytes can also be mobilized to different tissues in response to stress (Ellis et al., 2011), and return to baseline levels quickly when the organism is allowed to recover or acclimate (Pan et al., 2008; Delorme et al., in prep), therefore, the hemocyte concentration, viability and oxidative stress response at a given point in time can be highly variable and needs to be measured repeatedly over time. This was evident at timepoint 3 where mussel hemocyte concentrations had returned to baseline levels (e.g., similar to timepoint 1) in all treatments, despite their vastly different thermal history. A bias towards sampling more tolerant individuals (as we were sampling hemolymph and gill tissues from surviving individuals only) and the decrease in sample sizes available in the RH treatment at timepoint 3 (due to high mortalities) also may have decreased our sensitivity to detect differences between families and the RR and CTL treatments at this timepoint.

The bivalve immune system also responds to a variety of different stressors including pathogens, abiotic conditions and contaminants (Ellis et al., 2011). While the increase in mussel hemocyte concentration in this study was clearly due to an increase in seawater temperature, some of the more subtle differences in immune response (e.g., with respect to oxidative stress, TAC and hemocyte viability in different families even within the control treatment) may have also been due to additional factors that we did not measure. Although the experimental design of this study was very robust in terms of detecting family differences (e.g., individuals from all families were represented in every replicate tank, eliminating tank effects between families), the sampling process did not allow treatments to be sampled on the same day and control tanks were in a different room to RH and RR tanks. These factors may have also been driving some of the subtle immune responses observed (e.g., slight changes in food availability on different sampling days, or “room effects”).

However, the subtle differences in the immune response of family A compared with B and C in all temperature treatments are interesting. Family A was the best surviving family and overall, it appeared to have a different immune response to the other families with lower hemocyte concentration, and lower percentages of SO+ and dead hemocytes than the other families under a range of temperature conditions.

All mussels in our trial were the same age, but family A was much smaller in shell length compared with families B and C. Although there was no correlation between mussel size and time to death within each family in the RH treatment, the overall much smaller size of family A may have lowered its susceptibility to heat stress compared with the other families. Individuals with larger body mass have higher energy demands compared with smaller individuals, and energy requirements further increase with increasing seawater temperature (Sokolova et al., 2012). This may result in a loss of homeostasis and ultimately death in larger individuals that are unable to meet their increasing energy demands (Sebens, 2002). A significant size effect on thermal tolerance was observed in a recent study where larger juvenile *P. canaliculus* were less heat tolerant than their smaller counterparts of the same family, even in the more thermally tolerant families (Delorme et al., 2023). In our study, the differential survival and gene expression of families B and C that were a similar size does implicate the important role of other genetic factors on thermal tolerance.

### 4.2 Transcriptome and microbiome

Despite the overall high mapping rate of reads to the *de-novo* transcriptome, the BUSCO score indicated a relatively low completeness level compared to other Mytilid studies (see Moreira et al., 2018; Inoue et al., 2021). There are multiple reasons that could explain this low score including the presence of fragmented transcripts, low expression levels, alternative splicing and gene loss (Jauhal and Newcomb, 2021; Manni et al., 2021). Considering the relatively low N50 value of the assembly (1,655 bp), it is possible that BUSCO missed genes that were significantly fragmented (Jauhal and Newcomb, 2021). However, given the high mapping rate, it is also likely that many of the missing genes were either expressed at too low levels to be detected, loss in the green-lipped mussel, or that some of these genes underwent alternative splicing leading to gene variants with similarity levels below BUSCO threshold values. Since there is very limited genomic and transcriptomic information on this species, future molecular studies will be extremely valuable in elucidating these results.

Changes in seawater temperature, and the duration of change, were a strong driver of gene expression and gill microbiome composition in green-lipped mussels. Overall gene expression levels significantly shifted during thermal stress and when returned to ambient temperatures, expression patterns became non-significantly different from control treatments. A similar response was seen in the microbiome with beta-diversity shifting during the thermal stress experiment and with significant differences between families at the beginning of the experiment, but this difference became non-significant towards the end. At the gene level, numbers of DEGs in mussels were also strongly influenced by seawater temperature. However, GO terms affected during the sustained increase in water temperature were not necessarily the same as those impacted during recovery.

Specific genes that were upregulated by the treatments were more variable between families but DEGs that were common to all families and demonstrated expression patterns that responded and were induced by the treatments (i.e., reduced to baseline under ambient conditions) included those identified as heat shock proteins (HSPs) and other molecular chaperones (e.g., GRPEL1, HSP90B1, HSPE1, PPIB), immune response genes (e.g., AIF1, CTSC, TOLL8), apoptosis markers (e.g., CASP9, FNTA), and metabolism and oxidative stress markers (e.g., AHCY, CRYAB, PPIF). Molecular chaperones, including HSPs and others, are highly conserved proteins that are involved in the folding and transport of proteins (Craig et al., 1993). HSPs are known to be induced by stressful conditions and are important for recovery and survival—even small inductions have been associated with stress resistance, life span and fecundity (Sørensen et al., 2003). Because of this, HSPs have commonly been used as biomarkers of environmental stress (Sørensen et al., 2003). For example, HSP70 genes have shown to be upregulated in juvenile *P. canaliculus* after exposure to an acute thermal stress (Delorme et al., 2020), and in a recent study, the HSP70 gene was upregulated differently among families of subadult *P. canaliculus*, also correlating with their thermal tolerance (Delorme et al., 2023). In the present study, HSPs and other chaperone genes showed promise as biomarkers of stress in *P. canaliculus*.

Environmental stress is also known to impact the immunity of marine organisms and previous studies have demonstrated an immune gene response in bivalves to abiotic stressors (Mao et al., 2022). Immune response can be activated to increase defense and survivability during adverse conditions. Toxic effects, such as DNA damage or oxidative stress, can be induced by environmental stressors and are mediated by processes such as regulation of apoptosis (programmed cell death) and redox signaling (Franco et al., 2009). The gene-level immune response of mussels to thermal stress and recovery was much stronger than the more subtle changes observed using flow cytometry. This may be related to the kinetics of the responses; in another recent study hemolymph cell viability and oxidative stress (measured using flow cytometry) in heat-stressed *P. canaliculus* returned to baseline within 3 h after heat-stress, while HSP70 transcript-level remained elevated for days afterwards (Delorme et al., in prep). This suggests that gene-level responses may be easier to detect as they may remain detectable for longer durations in stressed individuals.

Genes and gene pathways associated with immune response, apoptotic cell death and metabolism/oxidative stress were commonly upregulated across all families during the thermal stress experiment. Interestingly, taxon from the microbiome analyses identified as putative pathogenic bacteria, including *Vibrio* spp. showed an increase in the RH treatments. Potentially the immune response in the mussels was stimulated by abiotic stress and might have made them more susceptible to pathogens [Azizan et al., (under review)]. Biomarkers that can measure the response of mussels to multi-stressors are useful to determine real-world resilience in a changing environment (Dahlhoff, 2004; Parisi et al., 2017; Ericson et al., 2022). Our group recently developed reverse-transcription quantitative polymerase chain reaction assays (RT-qPCR) for *P. canaliculus* targeting the expression of genes involved in oxidative stress, xenobiotic transfer, membrane transportation, cellular and DNA response/repair, and endocrine disruption (Baettig et al., 2023b). Significant modulation of genes occurred following exposure to common environmental pollutants, demonstrating the potential power of molecular biomarkers. However, the RT-qPCR assays developed were dependent on *a priori* knowledge from genomic resources from other mussel species. The assembly and characterization of the *P. canaliculus* transcriptome, specific to heat stress, in this study now enables identification of impacted gene pathways and the development of potential biomarkers that was not previously possible.

Similarly to the hemolymph immune response assays, transcriptome analyses yielded that family A was more distinct from families B and C. Family C had substantially more genes impacted by temperature treatment and timepoint than the A and especially family B (which had very little genes/pathways that responded to the treatments). Family C also had the highest number of differentially expressed pathways, followed by family A, while family B had none for either heat-stress treatment. After recovery, family C remained more impacted than the other families, with several pathways being suppressed compared to controls. In the families that had most resilience to heat stress (A and C), pathways related to immune response, protein folding, membrane function, ion binding and catalytic activity were upregulated. The microbiome of family A was also significantly different from families B and C at the beginning of the experiment. This difference was reduced at the end of the experiment, with the microbiome of all families converging and an overall increase in microbial richness in all families. A recent study describing the core microbiome of *P. canaliculus* demonstrated the strong association between the circulatory tissues, such as gills, and surrounding seawater environment (Li et al., 2022). It is likely that some of the microbiome shifts seen in this study are the result of the mussel families existing in the same seawater system and sharing microbes, and as a result no significant interaction between families and treatment was detected. However, microbiome diversity clearly showed a shift in response to temperature treatments. By the end of the experiment, the microbiome of RH mussels was extremely different to control and RR mussels, where the dominant bacterial family Gammaproteobacteria had been replaced by Bacteroidia and the families *Campylobacter* and the Vibrionaceae also increased. Microorganisms such as *Vibrio* and *Campylobacter* spp. have been found in raw and undercooked shellfish and have been associated with human illness (Ripabelli et al., 1999) and this study suggests that warmer sea temperatures could increase the number of illnesses globally.

### 4.3 Further development of non-lethal, rapid, diagnostic biomarkers and use in breeding programmes

Interest in non-lethal molecular diagnostics of stress is growing, as such an approach increases animal welfare, allows for repeated survey of individuals, and links gene expression to fitness following exposure to stress (Veldhoen et al., 2014; Czypionka et al., 2015; Jeffries et al., 2021; Hechler et al., 2022). The present study has unveiled promising insights into the stress response of selectively bred New Zealand green-lipped mussels to elevated seawater temperatures. Notably, we observed significant up or downregulation of genes related to stress response and related processes under increased temperature, indicating the mussels’ capacity to mount specific molecular responses to thermal challenges. Leveraging these findings, enables the potential for developing a suite of targeted assays, using highly specific techniques such as quantitative or droplet digital PCR (qPCR or ddPCR), based on a selection of candidate genes identified in this study that can serve as non-lethal, rapid diagnostic biomarkers in farmed mussels. Additional research on links between expression of targeted genes and protein synthesis is necessary, to further inform how these genes are linked to specific physiological responses.

The adoption of these targeted PCR-based assays as diagnostic tools in breeding programmes holds significant potential for advancing mussel aquaculture resilience in the face of ocean warming. By selectively breeding individuals with superior stress response capabilities, we can enhance the overall thermotolerance of cultured mussel stocks, leading to increased survival and improved health under elevated seawater temperatures. In conclusion, our study sets the stage for the development and utilization of non-lethal, rapid diagnostic biomarkers based on stress gene expression patterns observed in selectively bred mussels. The integration of these targeted PCR assays into breeding programs has the potential to revolutionize the industry by enabling early identification of stress-susceptible individuals and facilitating the selection of candidates with enhanced resilience to ocean warming. This approach represents a significant step towards building more robust and adaptable mussel populations, ensuring the sustainability and productivity of aquaculture in a changing climate.

## Data Availability

The genomics datasets presented in this article are not readily available, because green-lipped mussels are a taonga species of significant cultural importance to New Zealand Māori. Accordingly, all data relating to this manuscript are stored here on the Aotearoa Genomic Data Repository doi.org/10.57748/S5A6-7V40. It can be requested via the repository website, and the decision-making process regarding whether the data is shared will be made by those holding kaitiakitanga (guardianship) over the data.
