# Supplementary material for: Differential responses of selectively bred mussels (Perna canaliculus) to heat stress—survival, immunology, gene expression and microbiome diversity
Source: Front Physiol. 2024 Feb 15;14:1265879. doi: 10.3389/fphys.2023.1265879 (PMC10902150; doi:10.3389/fphys.2023.1265879)
Supplement: Supplementary file 2 [file Table2.docx]

Table S5 | Gene acronyms and full names

| ACTA2 | Actin Alpha 2, Smooth Muscle |
| --- | --- |
| AHCY | Adenosylhomocysteinase |
| AIF1 | Allograft Inflammatory Factor 1 |
| ARRDC3 | Arrestin Domain Containing 3 |
| ATP6 | ATP Synthase Membrane Subunit 6 |
| CASP9 | Caspase 9 |
| CEBPG | CCAAT Enhancer Binding Protein Gamma |
| CRYAB | Crystallin Alpha B |
| CTSC | Cathepsin C |
| CYP1 | Cytochrome P450 Family 1 |
| DNAJA1 | DnaJ Heat Shock Protein Family (Hsp40) Member A1 |
| DSP1 | Desmoplakin Family 1 |
| FCER2 | Fc Epsilon Receptor II |
| FNTA | Farnesyltransferase, CAAX Box, Alpha |
| GRPEL1 | GrpE Like 1, Mitochondrial |
| HNRNPD | Heterogeneous Nuclear Ribonucleoprotein D |
| HNRNPK | Heterogeneous Nuclear Ribonucleoprotein K |
| HSP70 | Heat Shock Protein 70 |
| HSP90B1 | Heat Shock Protein 90 Beta Family Member 1 |
| HSPA9 | Heat Shock Protein Family A (Hsp70) Member 9 |
| HSPE1 | Heat Shock Protein Family E (Hsp10) Member 1 |
| MAX | MYC Associated Factor X |
| MYC | MYC Proto-Oncogene, BHLH Transcription Factor |
| NCL | Nucleolin |
| PDIA3 | Protein Disulfide Isomerase Family A Member 3 |
| PFDN4 | Prefoldin Subunit 4 |
| PIK3R4 | Phosphoinositide-3-Kinase Regulatory Subunit 4 |
| PPIB | Peptidylprolyl Isomerase B |
| PPD3 | Protein phosphatase D3 |
| PPIF | Peptidylprolyl Isomerase F |
| PRKCSH | Protein Kinase C Substrate 80K-H |
| PTGS2 | Prostaglandin-Endoperoxide Synthase 2 |
| RBM15 | RNA Binding Motif Protein 15 |
| rRNA | Ribosomal RNA |
| SNX9 | Sorting Nexin 9 |
| STIP1 | Stress Induced Phosphoprotein 1 |
| TOLL8 | Tollo 8 |
