# Supplementary material for: Differential responses of selectively bred mussels (Perna canaliculus) to heat stress—survival, immunology, gene expression and microbiome diversity
Source: Front Physiol. 2024 Feb 15;14:1265879. doi: 10.3389/fphys.2023.1265879 (PMC10902150; doi:10.3389/fphys.2023.1265879)
Supplement: Supplementary file 6 [file DataSheet1.docx]

Supplementary material

| Table S1. Sample sizes (n) of mussels sampled for hemolymph within each family and temperature treatment at each timepoint | | | |
| --- | --- | --- | --- |
| **Timepoint 1 (T1)** | | | |
|  | Control (CTL) | Ramp and Recover (RR) | Ramp and Hold (RH) |
| Family A | 16 | 16 | 16 |
| Family B | 16 | 16 | 16 |
| Family C | 16 | 16 | 16 |
| **Timepoint 2 (T2)** | | | |
|  | Control (CTL) | Ramp and Recover (RR) | Ramp and Hold (RH) |
| Family A | 16 | 15 | 16 |
| Family B | 15 | 16 | 16 |
| Family C | 16 | 16 | 16 |
|  |  |  |  |
| **Timepoint 3 (T3)** | | | |
|  | Control (CTL) | Ramp and Recover (RR) | Ramp and Hold (RH) |
| Family A | 16 | 15 | 8 |
| Family B | 15 | 15 | 3 |
| Family C | 16 | 16 | 5 |

| Table S2. Log-rank test pairwise comparisons for each temperature*family treatment | | | | | | | | | |
| --- | --- | --- | --- | --- | --- | --- | --- | --- | --- |
|  | Family A  Control | Family B Control | Family C Control | Family A Ramp + Recover | Family B Ramp + Recover | Family C Ramp + Recover | Family A Ramp + Hold | Family B Ramp + Hold | Family C Ramp + Hold |
| Family B Control | 0.237 |  |  |  |  |  |  |  |  |
| Family C Control | 1.000 | 0.225 |  |  |  |  |  |  |  |
| Family A Ramp + Recover | 0.628 | 0.152 | 0.628 |  |  |  |  |  |  |
| Family B Ramp + Recover | 1.000 | **0.024** | 1.000 | 0.408 |  |  |  |  |  |
| Family C Ramp + Recover | 0.535 | 0.317 | 0.531 | 0.628 | 0.225 |  |  |  |  |
| Family A Ramp + Hold | **<0.001** | **<0.001** | **<0.001** | **<0.001** | **<0.001** | **<0.001** |  |  |  |
| Family B Ramp + Hold | **<0.001** | **<0.001** | **<0.001** | **<0.001** | **<0.001** | **<0.001** | **<0.001** |  |  |
| Family C Ramp + Hold | **<0.001** | **<0.001** | **<0.001** | **<0.001** | **<0.001** | **<0.001** | **0.001** | **<0.001** |  |


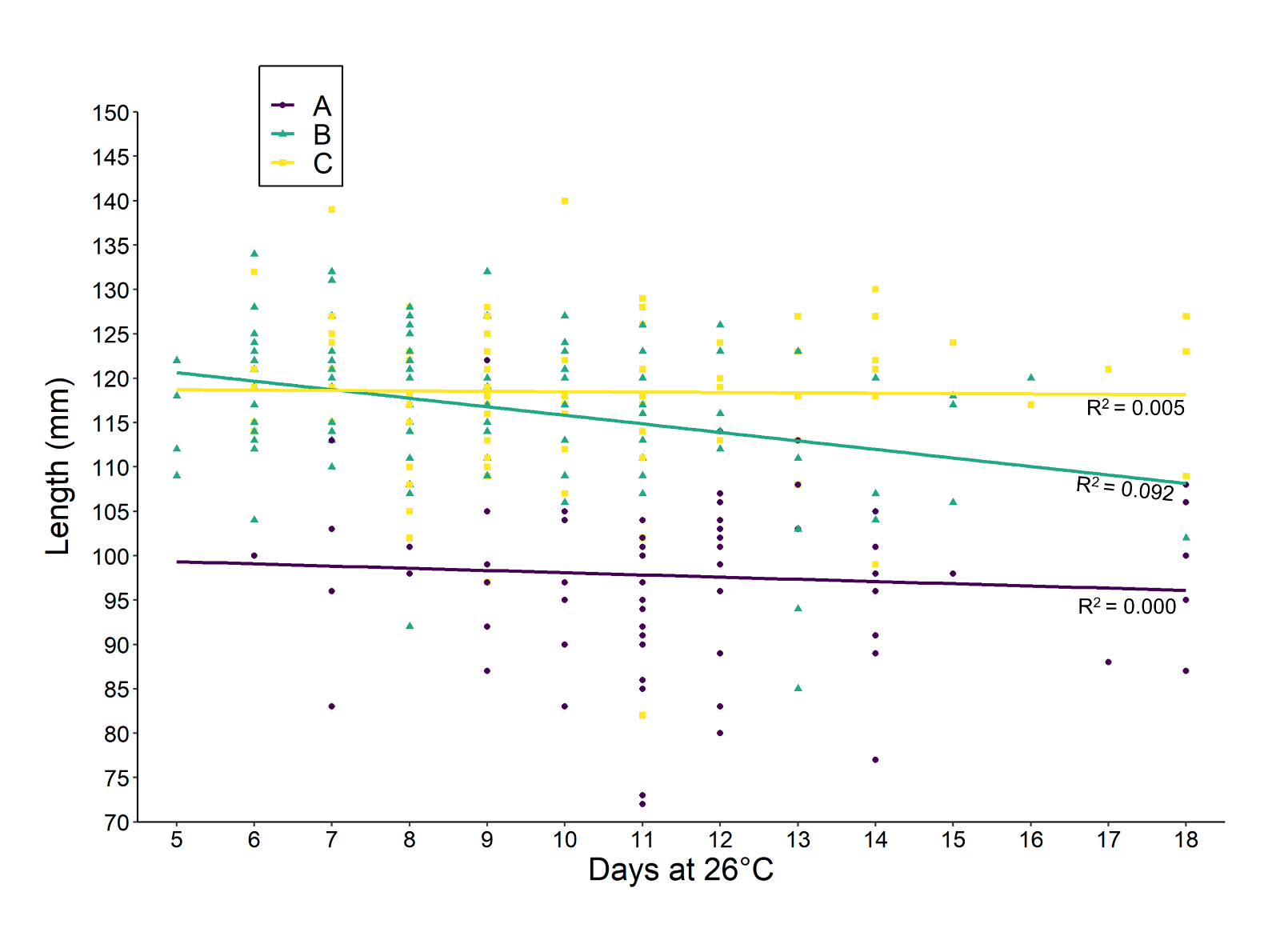


Figure S1. Mussel shell length (mm) plotted against the period of exposure to 26 °C before the mussel died. Regression lines are shown for each family (A, B and C).

Figure S2. Potential heat stress gene expression biomarkers, selected from differentially expressed genes (DEGs) that were common to all families from the Ramp and Hold treatment. Responses to the Ramp and Hold and Ramp and Recovery treatments are shown. Gene functions are shown in Table S4.

Figure S3. Microbial Shannon diversity per green-lipped mussel family and sampling point for the control samples (A), and per treatment and sampling point (B). Significant differences between groups are indicated by horizontal lines and stars over the relevant boxplots. C = Control, RR = Ramp and recover, RH = Ramp and hold, T = Timepoint, * = p<=0.05, ** = p<=0.01, *** = p<=0.001.

Figure S4. Bacterial taxa found significantly differentially abundant at sampling point 3 between Controls (CTL) and Ramp and hold (A) and between Controls and Ramp and Recover (B). Taxa identified as putative pathogens are highlighted in bold and followed by an asterisk.
